# Imaging of cancer of unknown primary: a systematic literature review of the past, present, and future

**DOI:** 10.1093/bjr/tqaf039

**Published:** 2025-03-21

**Authors:** Sajjad Rostami, Hannah W Stutterheim, Olga Maxouri, Jeroen R J Willemse, Diana Ivonne Rodríguez Sánchez, Winnie Schats, Larissa W van Golen, Marieke A Vollebergh, Zing Cheung, Wouter V Vogel, Serena Marchetti, Petur Snaebjornsson, Max J Lahaye, Doenja M J Lambregts, Zuhir Bodalal, Regina G H Beets-Tan

**Affiliations:** Department of Radiology, The Netherlands Cancer Institute, 1066 CX Amsterdam, The Netherlands; GROW Research Institute for Oncology and Reproduction, Maastricht University, 6211 LK Maastricht, The Netherlands; Department of Radiology, The Netherlands Cancer Institute, 1066 CX Amsterdam, The Netherlands; Department of Radiology, The Netherlands Cancer Institute, 1066 CX Amsterdam, The Netherlands; GROW Research Institute for Oncology and Reproduction, Maastricht University, 6211 LK Maastricht, The Netherlands; Department of Radiology, The Netherlands Cancer Institute, 1066 CX Amsterdam, The Netherlands; GROW Research Institute for Oncology and Reproduction, Maastricht University, 6211 LK Maastricht, The Netherlands; Department of Radiology, The Netherlands Cancer Institute, 1066 CX Amsterdam, The Netherlands; GROW Research Institute for Oncology and Reproduction, Maastricht University, 6211 LK Maastricht, The Netherlands; Scientific Information Service, The Netherlands Cancer Institute, 1066 CX Amsterdam, The Netherlands; Department of Nuclear Medicine, The Netherlands Cancer Institute, 1066 CX Amsterdam, The Netherlands; Department of Gastrointestinal Oncology, The Netherlands Cancer Institute, 1066 CX Amsterdam, The Netherlands; Department of Nuclear Medicine, The Netherlands Cancer Institute, 1066 CX Amsterdam, The Netherlands; Department of Nuclear Medicine, The Netherlands Cancer Institute, 1066 CX Amsterdam, The Netherlands; Department of Radiation Oncology, The Netherlands Cancer Institute, 1066 CX Amsterdam, The Netherlands; Department of Medical Oncology and Clinical Pharmacology, The Netherlands Cancer Institute, 1066 CX Amsterdam, The Netherlands; Department of Pathology, The Netherlands Cancer Institute, 1066 CX Amsterdam, The Netherlands; Faculty of Medicine, University of Iceland, 101 Reykjavik, Iceland; Department of Radiology, The Netherlands Cancer Institute, 1066 CX Amsterdam, The Netherlands; GROW Research Institute for Oncology and Reproduction, Maastricht University, 6211 LK Maastricht, The Netherlands; Department of Radiology, The Netherlands Cancer Institute, 1066 CX Amsterdam, The Netherlands; GROW Research Institute for Oncology and Reproduction, Maastricht University, 6211 LK Maastricht, The Netherlands; Department of Radiology, The Netherlands Cancer Institute, 1066 CX Amsterdam, The Netherlands; GROW Research Institute for Oncology and Reproduction, Maastricht University, 6211 LK Maastricht, The Netherlands; GROW Research Institute for Oncology and Reproduction, Maastricht University, 6211 LK Maastricht, The Netherlands; Faculty of Health Sciences, University of Southern Denmark, 5230 Odense M, Denmark; The Netherlands Cancer Institute, 1066 CX Amsterdam, The Netherlands

**Keywords:** cancer of unknown primary, medical imaging, CT, MRI, PET, FDG, FAPI, radiomics, artificial intelligence

## Abstract

**Objectives:**

To evaluate the evolution and current diagnostic capabilities of medical imaging in cancer of unknown primary (CUP) and explore promising technologies for enhancing diagnostic precision.

**Methods:**

A comprehensive literature search was conducted across MEDLINE, Embase, and Scopus in March 2023 (updated in August 2024) to identify original articles focusing on CUP imaging. Two reviewers independently selected articles and extracted data. Quality assessment was performed using QUADAS-2 and Radiomics Quality Score. Given the variability in study designs, imaging techniques, and reported outcomes, a narrative synthesis was performed. Subgroup analyses compared detection rates across modalities.

**Results:**

From 4760 de-duplicated search results, 140 original articles were included. Early CUP imaging relied on 2D modalities with notable diagnostic limitations. Modern 3D modalities have risen in prominence, though mammography and ultrasound remain in CUP guidelines. Implementing CT and MRI significantly improved primary tumour detection and disease characterization. CT is fundamental for CUP evaluation, and MRI offers superior soft tissue resolution, effective for detecting occult breast cancer, head and neck primaries, and suspected abdominopelvic neoplasms. FDG-PET/CT showed varying primary detection capabilities, adding value in identifying lesions/metastases missed by other modalities, essential for confirming locoregional treatment strategies. Emerging technologies for CUP imaging include whole-body MRI, FAPI-PET/CT, and AI/radiomics.

**Conclusions:**

Advancements in imaging have improved the diagnostic workup for CUP. Innovative approaches show potential for further improvement in diagnostic accuracy.

**Advances in knowledge:**

This study provides a comprehensive overview of CUP imaging and introduces emerging modalities that could boost diagnostic accuracy.

**Prospero registration:**

CRD42023453393.

## Introduction

Cancer of unknown primary (CUP) describes a heterogeneous group of cancers, characterized by the presence of metastatic disease with an unidentified primary tumour despite thorough diagnostic efforts.^1–3^ CUP constitutes approximately 2%-5% of all newly diagnosed cancers globally, but disproportionately accounts for 8% of cancer-related deaths due to its high mortality rate.^3–5^ The elusive nature of CUP presents significant clinical consequences, complicating treatment strategies and leaving both patients and clinicians uncertain of the best course of action. Consequently, the long-term prognosis of these patients is impacted.^5^

Patients with CUP who do not fit into recognized favourable risk subgroups often receive suboptimal empirical treatments (or no treatment at all). The favourable subsets, representing only 10%-20% of CUP cases, may benefit from tailored treatments analogous to those for cancers with known origins, potentially achieving comparable survival outcomes.^3^^,^^5^^,^^6^ In contrast, the majority (80%-90%) of CUP patients are classified within the unfavourable risk group, where research-based prognostic models attempt to stratify patients into poorer- and better-prognosis subgroups, with median survival times of 3.9 and 11.7 months, respectively.^7^ Overall, the clinical outcome of CUP is poor, with only 16%-20% of patients surviving beyond the first year.^3^^,^^8^

The challenge of unidentifiable primary tumours is not a new phenomenon; it dates back to the earliest days of cancer diagnosis. However, only in the 1940s was CUP first recognized as a distinct clinical entity in oncology, mainly due to the limitations of diagnostic modalities at the time.^9^ Since then, the definition of CUP has evolved in parallel with advancements in diagnostic technologies, contributing to a decline in reported CUP cases as more primary tumours are detected.^10–12^ Despite these advancements, some primary tumours remain undetectable with current diagnostic workups, maintaining CUP as a relevant clinical diagnosis. The American Cancer Society reports a notable trend in CUP incidence in the United States, where the estimated annual number of CUP patients registered with healthcare facilities remained stable, despite CUP forming a decreasing percentage of overall cancer diagnoses over the past 55 years.^4^

Within the diagnostic framework for CUP, medical imaging is a fundamental component, as highlighted by several clinical guidelines.^1–3^^,^^13^ Imaging plays a key role not only in the search for the primary tumour, but also in providing comprehensive information about the extent and nature of the disease, which is essential for effective treatment planning and follow-up.^14^ The evolution of medical imaging has strongly influenced the diagnosis/management of CUP. For instance, 18F-fluorodeoxyglucose (FDG)-PET/CT has been extensively studied and shown advantages over conventional imaging in detecting primary head and neck (H&N) tumours in CUP.^15^^,^^16^ Similarly, advances in MRI, including diffusion-weighted imaging (DWI), have enhanced the ability to detect and characterize lesions and guide biopsies, particularly in breast cancer, renal cell carcinoma, and liver metastases.^17–19^ Moreover, integrating artificial intelligence (AI) and machine learning algorithms in image analysis holds promise for further refining the diagnostic accuracy and prognostic assessment of CUP. These technologies can analyse vast amounts of imaging data to identify patterns and anomalies that might elude human observers, possibly assisting in uncovering primary sites and providing personalized treatment options. As imaging technologies advance, their impact on CUP diagnosis and treatment will likely grow.

In this review, we aim to explore the role of medical imaging in the CUP clinical workup and provide a narrative overview of its historical, current, and potential future impacts. This review can serve as a comprehensive educational resource for researchers, radiologists, and members of CUP multidisciplinary teams, and reduce the need to navigate fragmented literature for future studies on CUP imaging.

## Methods

### Guideline

This systematic review is reported according to the Preferred Reporting Items for Systematic reviews and Meta-Analyses (PRISMA) guidelines.^20^ Additionally, it adheres to the Guidance on the Conduct of Narrative Synthesis in Systematic Reviews and the Synthesis Without Meta-analysis (SWiM) reporting criteria.^21^^,^^22^ The review protocol was registered pre-inception with the International Prospective Register of Systematic Reviews (PROSPERO) under the registration ID CRD42023453393.^23^

### Literature search

A comprehensive search of literature was conducted across MEDLINE, Embase, and Scopus databases in March 2023 (updated in August 2024) to identify published studies on imaging of CUP. The search strategy used a combination of terms related to different medical imaging modalities and CUP, accounting for different naming conventions. The literature search for this review was restricted to articles written in English, with no limitations regarding publication date or study design. Specific search terms and strategies are detailed in Table S1.

### Study eligibility and selection

Studies were included in this review only if they were directly related to the use of medical imaging in CUP. Excluded materials included abstracts, case reports, letters, editorials, comments, and reviews. In line with the most recently published ESMO (European Society of Medical Oncology) guideline for CUP, we excluded studies on sarcomas, melanomas, germ cell tumours, hematological malignancies, and neuroendocrine tumours.^3^ Two reviewers (S.R., Z.B.) independently screened the titles and abstracts of all the retrieved records. Full-text versions of shortlisted articles were assessed for eligibility (S.R., H.S.). Disagreements were resolved by consensus, and a third reviewer (Z.B.) was consulted to reach a decision if necessary.

### Data extraction and quality assessment

Aligned with the review question, data extraction focused on the study characteristics, patient characteristics, baseline imaging diagnostics, examined imaging modalities, diagnostic performances, and relevant technical information such as imaging devices, tracers, and sequences. Depending on the nature of methodology, each included study was assessed for quality and risk of bias using either the latest version of the Quality Assessment of Diagnostic Accuracy Studies (QUADAS-2) or the Radiomics Quality Score (RQS).^24^^,^^25^

### Synthesis of results

Since a meta-analysis of effect estimates was not deemed feasible (considering the heterogeneous nature of available data) and we primarily sought to provide a descriptive overview, a narrative synthesis was used to describe the role of imaging in CUP diagnostic workflows over time. Criteria for including studies in the narrative synthesis were based on study design, risk of bias assessments, and relevance to the review question. Subgroup analyses compared diagnostic performances across imaging modalities. The comparative analyses aimed to map the potential causal effect of various advancements in imaging on improved primary tumour detection rates (DRs), and provide insights into emerging CUP diagnostic trends.

## Results

### Literature search and study selection

**Figure 1.**
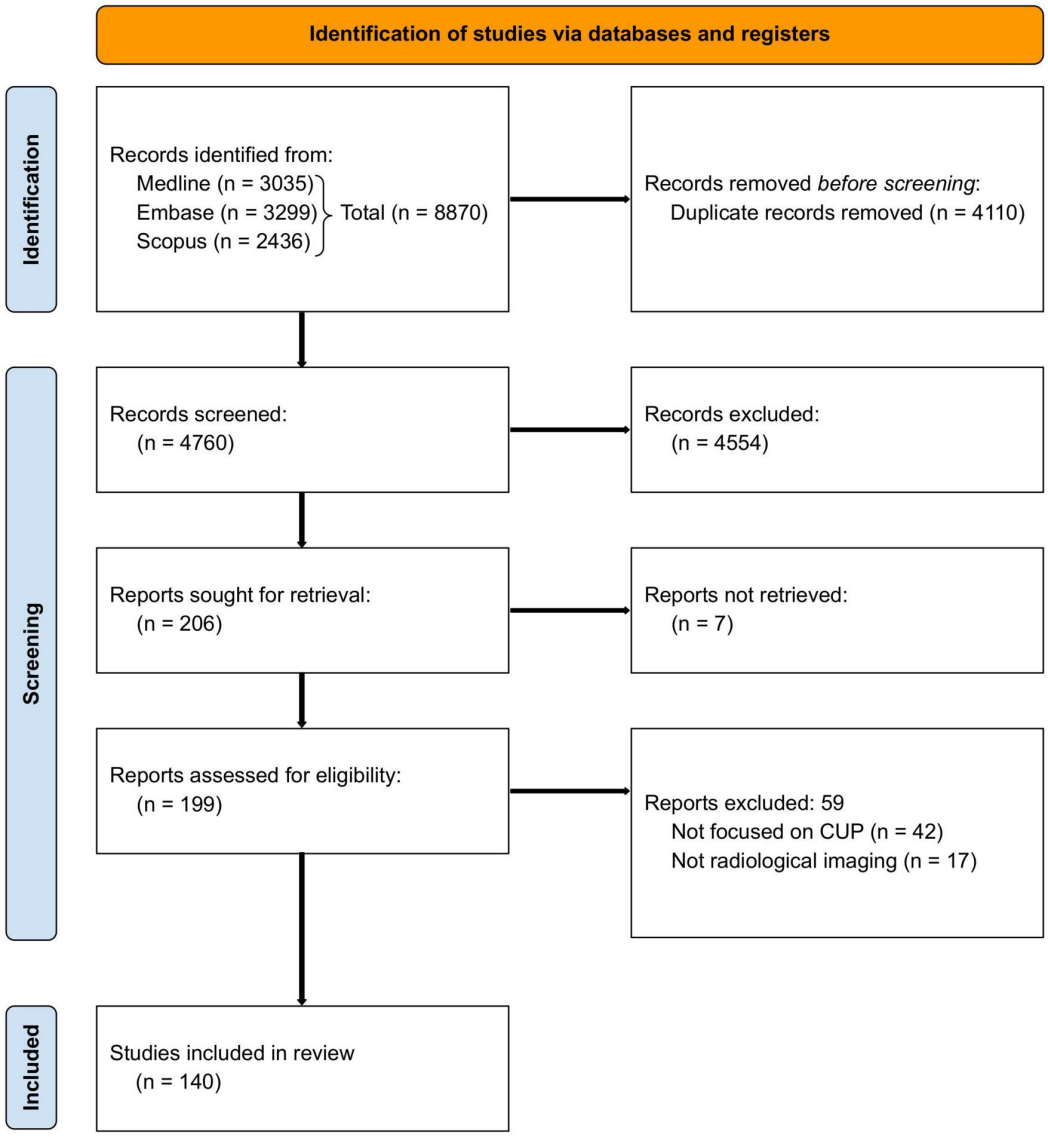
PRISMA flowchart for the study inclusion process. This flowchart depicts the process of identifying, screening, and selecting studies for inclusion in this systematic review.

An initial search of literature across MEDLINE, Embase, and Scopus, followed by de-duplication, resulted in 4760 unique records, of which ultimately 140 studies were selected for this systematic review. A flow diagram of the study selection process is provided in Figure 1.

### Study characteristics

**Figure 2.**
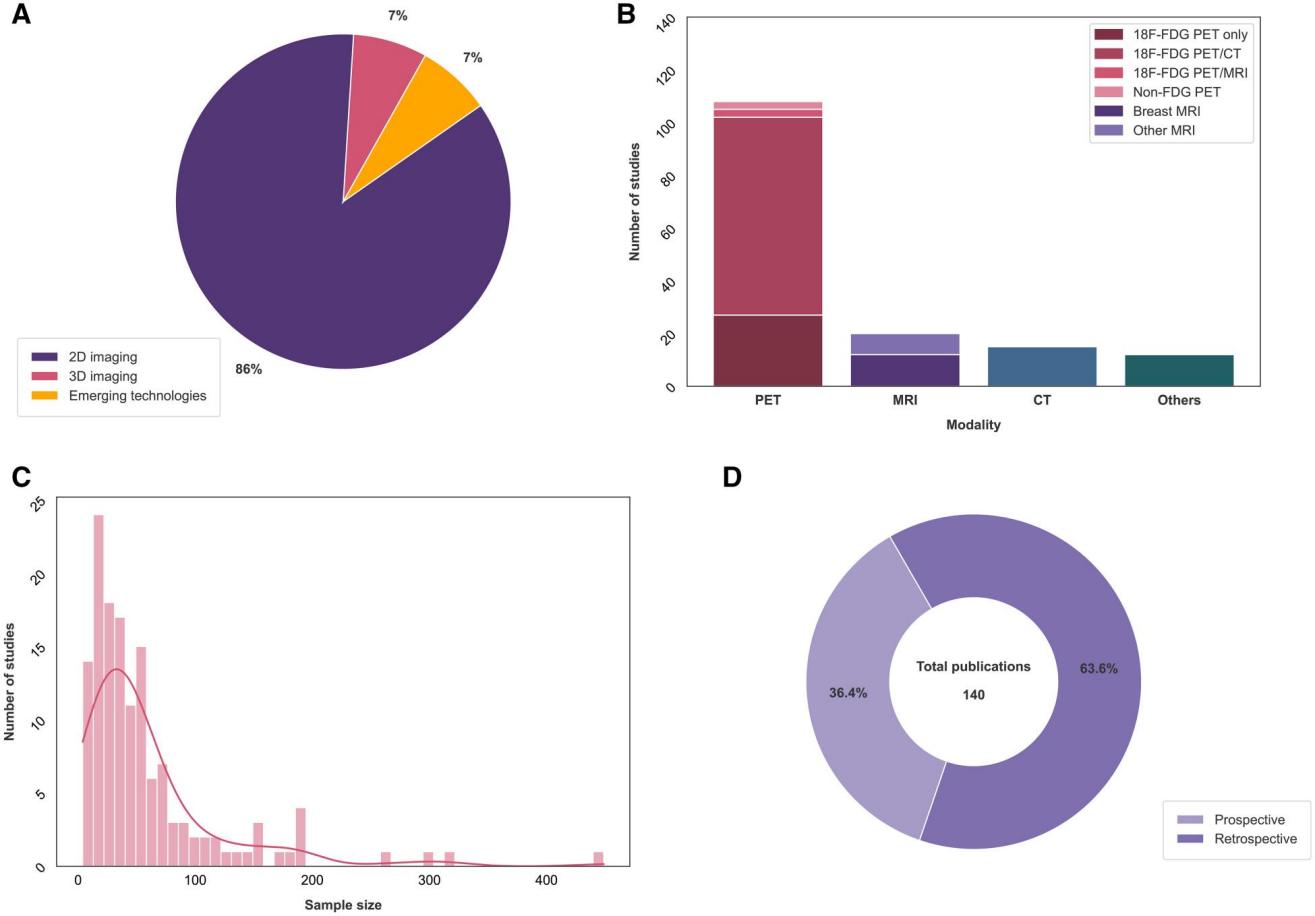
Graphical overview of the study characteristics. (A) Proportional representation of the included studies across 2D imaging, 3D imaging and emerging technologies. (B) Quantitative distribution of studies per modality (PET, MRI, CT, and others). (C) Histogram illustrating the distribution of study sample sizes of the included studies. (D) Percentage of prospective versus retrospective studies within the total 140 publications.

The studies reviewed cover the period from 1976 to 2024, with 10 studies focusing on 2D imaging (including ultrasound), 120 on 3D imaging (CT, MRI, PET), and 10 on emerging technologies. 18F-FDG-PET and PET/CT constituted approximately 71% (99/140) of the literature on CUP imaging. The study populations for all the included records fall within the range of 4-449 patients, with a median of 39 and an interquartile range (IQR) of 45 patients. Figure 2 illustrates a graphical overview of the study characteristics.

### Quality assessment

**Figure 3.**
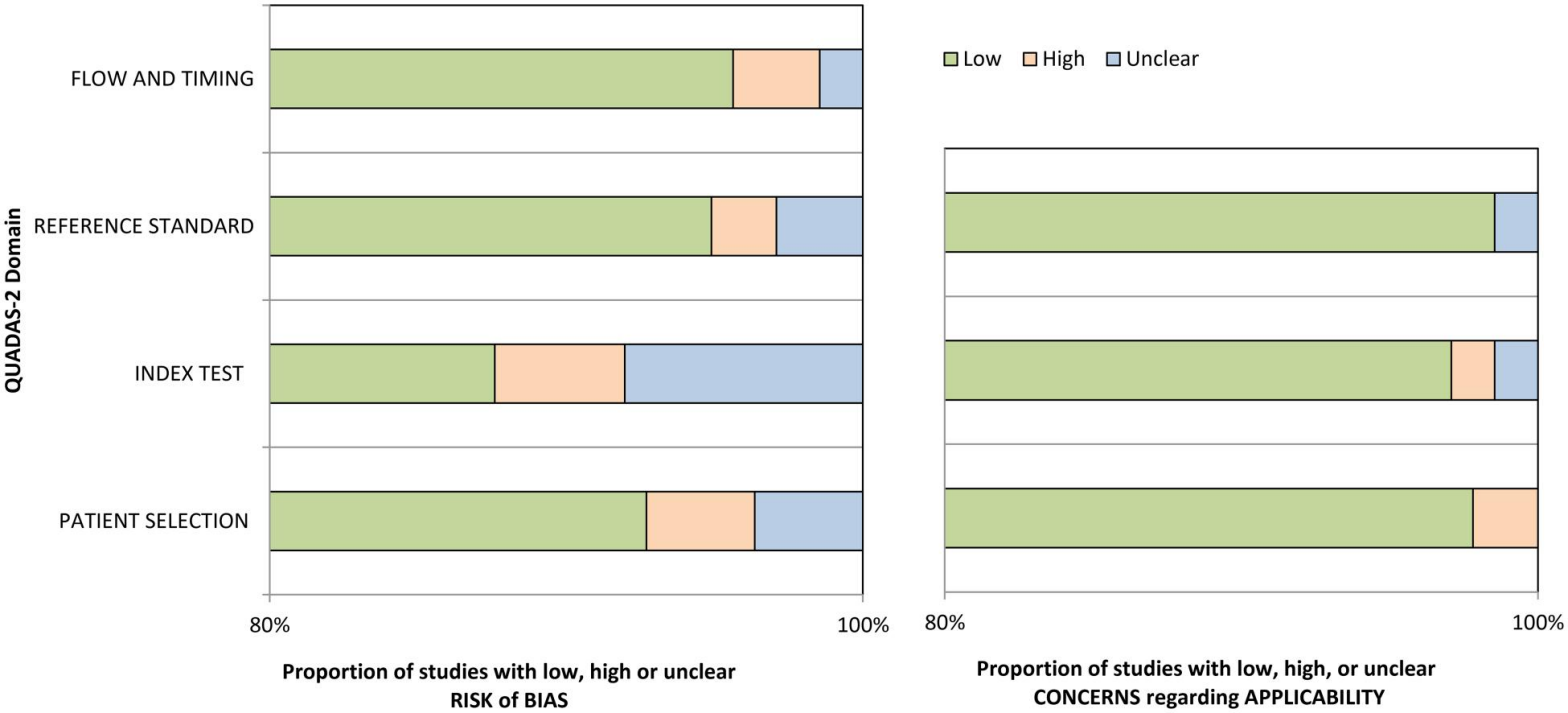
Risk of bias and applicability concerns. A summary of the risk of bias, applicability concerns, and quality assessment of the 137 included studies evaluated via the QUADAS-2 tool.

The quality and methodological bias of 137 of the 140 included studies were assessed using the QUADAS-2 assessment tool. The index test and patient selection domains mainly contributed to a higher risk of bias and raised applicability concerns in these studies, as shown in Figure 3. The remaining three studies that implemented radiomics in their methodology were assessed using the RQS. Each of these studies received an identical RQS of 13, indicating a moderate level of quality.

## Discussion

### The role of imaging in CUP

**Figure 4.**
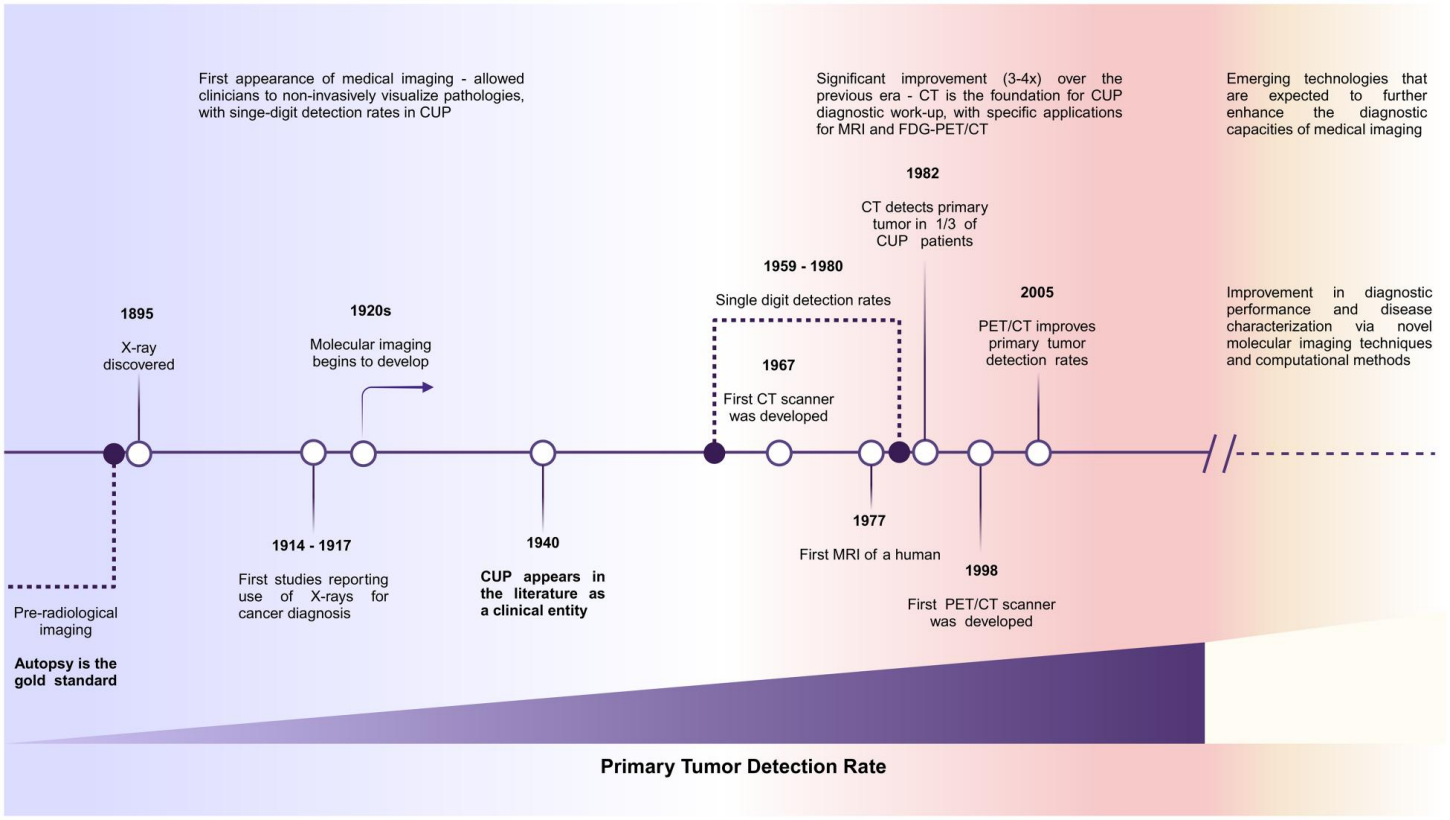
Timeline of the evolution of medical imaging in regard to cancer of unknown primary. This timeline showcases the key milestones in imaging technologies and their implications for cancer of unknown primary.

An overview of the major landmarks in the evolution of medical imaging and their impact on CUP is outlined in Figure 4; the pearls and pitfalls of the different modalities are furthermore highlighted in Table 1. A detailed discussion of different modalities is provided in the following sections.

**Table 1.** Overview of key imaging modalities in the evaluation of cancer of unknown primary.

Imaging modality/era	Performance	Pearls	Challenges	Current role in CUP
2D imaging (X-ray, ultrasound)	Generally low detection rates for primary tumours (often single-digit)	Accessibility, low cost, suitable for triaging and screening	Low-resolution imaging, risk of mislabeling lesions, observer-dependent	Mostly overshadowed by modern imaging; mammography and ultrasound remain relevant in specific contexts
CT	Improved primary tumour detection versus conventional imaging	Rapid, widely accessible, and provides comprehensive anatomical coverage	Poorer soft-tissue contrast than MRI, may miss morphologically occult lesions	Cornerstone for initial assessment and staging, mandatory before labeling patients as CUP
MRI	High detection rates in select contexts (occult breast and head & neck cancer)	Favourable soft tissue resolution, combines anatomic and functional imaging (e.g., DWI)	Less accessible than CT, longer scan times, not ideal for suspected occult lung cancers	Recommended alongside CT in specific scenarios (suspected head & neck primaries, brain metastases, pelvic neoplasms)
FDG-PET(/CT)	Variable detection rates (5%-75%, pooled ∼41%), effective at staging, influences therapeutic decisions (∼24%-69% of cases)	Metabolic assessment beyond structural imaging, guides targeted biopsies by identifying metabolically active lesions	Mixed results in primary tumour detection, false positives/negatives	Valuable in select contexts (e.g., ruling out distant metastases, guiding biopsies in cervical CUP)

### 2D imaging

#### X-ray, fluoroscopy, and ultrasound

Only a few (mainly older) studies have specifically investigated CUP using 2D imaging techniques. One notable article, published in 1988 by Le Chevalier et al, reported a retrospective analysis of 302 autopsied patients with CUP presentations between 1959 and 1980. In this study, chest X-ray, the primary imaging modality at the time, identified a potential primary site in 32% of patients, however, they were confirmed true positives (TP) in only 10%. Upper gastrointestinal series revealed tumour-related findings in 12% of scans (one-third TP). Intravenous pyelography and barium enema demonstrated TP rates of 53% and 63%, respectively, although patient numbers were small (<20) in both groups. Other modalities, such as oral cholecystography and mammography, provided negligible additional diagnostic value in this study.^26^ As evidenced by several smaller studies, CUP imaging research from this period presented mainly single-digit detection rates.^27–32^

In a study focusing solely on patients with brain metastases of unknown origin, chest X-ray demonstrated better diagnostic value. Fifty-six patients (out of the 72 included) had positive findings for a suspected primary tumour in the lung, of which 84% were confirmed by histopathology.^33^ This improved detection rate can be partially attributed to the higher likelihood of brain metastases originating from lung cancer, increasing the pre-test diagnostic probability of CXR in this particular cohort.

Our review found only two studies specifically assessing ultrasound imaging in CUP. The first, by Osteen et al in 1978, indicated that gray-scale ultrasound provided new information (beyond what was found in physical examination) in five out of 27 cases. However, in eight others ultrasound proved misleading, since it falsely suggested the presence of retroperitoneal masses, resulting in unnecessary additional diagnostic workup.^27^ A more recent small study by Fakhry et al (*n* = 10) from 2014, focusing specifically on patients with H&N squamous cell CUP, showed that transcervical and intraoral ultrasound correctly identified the primary tumour in 70% of the cases after inconclusive FDG-PET/CT and H&N examinations.^34^

#### Early molecular imaging (scintigraphy)

Early molecular imaging techniques, including bone, liver, and thyroid scintigraphy scans, were the most commonly used imaging techniques in the early days of CUP imaging. Despite limited evidence, the existing literature reported the potential for primary tumour identification using these modalities in specific patient groups. In a study by Lentle et al, Ga-67 scintigraphy could correctly identify the primary tumour sites in 48% of suspected CUP cases.^35^ While bone scintigraphy was minimally valuable for primary tumour detection due to the rarity of primary bone tumours, they were helpful in screening for occult skeletal metastases.^27^

#### Summary of pearls and pitfalls of 2D imaging

A review of the CUP imaging literature from the early days of CUP diagnostics highlights several key points. Firstly, none of the articles provided a clear definition of CUP as a clinical entity. Without a shared understanding of what constitutes CUP, study results cannot be effectively consolidated or quantitatively/statistically analysed to draw conclusions. As a result, studies from this period had broad inclusion criteria, often resulting in overestimating the diagnostic capacity of a given modality. For instance, information derived from medical history and physical examination alone were reported to successfully indicate the primary tumour site in 10% of cases (yet these patients were classified as CUP).^36^ Even when a roentgenographic modality would suggest a potential primary tumour, the accuracy of that recommendation is often debateable (especially for abdominal malignancies). Due to low-resolution imaging and soft tissue complexity, primary tumours were sometimes overlooked, with metastases mistakenly labelled as primary tumours.^26^^,^^37^ For example, patients with metastatic pancreatic cancer and pulmonary metastases were repeatedly misdiagnosed as primary lung cancers.^38^ Le Chevalier et al’s post-autopsy study identified 13 instances of such mislabeling in their cohort of 302 patients.^26^ The suboptimal performance of conventional 2D imaging in anatomical areas with higher soft tissue complexity can also explain the disproportionate representation of subdiaphragmatic primary tumours among the final diagnoses of CUP patients in this period.^39^

Despite the limitations of early medical imaging modalities, they allowed for routine identification of primary tumours, leaving only a minority unidentified and classified as CUP. While 2D imaging has nowadays largely been overshadowed by modern 3D imaging techniques within the CUP guidelines, their accessibility and significance in rapid interpretation and screening of patients with potential malignancies remain substantial.

### 3D radiological imaging

The transition from 2D to 3D imaging marked a significant milestone in the evolution of medical imaging in general, as well as CUP, with the majority of CUP imaging research (∼86%) focusing on 3D imaging (Figure 2A). An overview of the key findings (primarily focused on primary tumour detection rates) of studies on CT and MRI is provided in Table 2. Further highlights are discussed below.

**Table 2.** Summary of included studies that reported primary tumour detection rates using CT and/or MRI in CUP.

Authors	Year	Modality	Study population	Primary tumour detection rate	Study type	Specific anatomical region
Karsell et al	1982	CT	98	DR = 32%	Retrospective	Mixed
Muraki et al	1984	CT	17	DR = 31%	Prospective	H&N
Tilanus-Linthorst et al	1997	MRI	4	DR = 100%	Prospective	Breast
Morris et al	1997	MRI	12	DR = 75%	Prospective	Breast
Stomper et al	1999	MRI	8	DR = 25%	Prospective	Breast
Schorn et al	1999	MRI	14	DR = 43%	Prospective	Breast
Orel et al	1999	MRI	22	DR = 86%	Retrospective	Breast
Henry-Tillman et al	1999	MRI	10	DR = 100%	Retrospective	Breast
Olson Jr et al	2000	MRI	40	DR = 70%	Prospective	Breast
Obdeijn et al	2000	MRI	31	DR = 35%	Prospective	Breast
Gutzeit et al	2005	CT	45	DR = 18%	Retrospective	Mixed
Freudenberg et al	2005	CT	21	DR = 23%	Retrospective	H&N
Buchanan et al	2005	MRI	69	DR = 48%	Retrospective	Breast
Nassenstein et al	2007	CT	39	DR = 13%	Prospective	H&N
Ko et al	2007	MRI	12	DR = 83%	Prospective	Breast
Lieberman et al	2008	MRI	16	DR = 81%	Retrospective	Breast
Gu et al	2008	DWI MRI	34	DR = 68%	Prospective	Mixed
Waltonen et al	2009	CT	183	DR = 10%	Retrospective	H&N
Roh et al	2009	CT	44	DR = 16%	Prospective	H&N
Møller et al	2012	CT	135	DR = 32%	Prospective	Extracervical
Lee et al	2015	CT * MRI	56	DR = 9% * DR = 23%	Prospective	H&N
Godeny et al	2016	MP MRI	38	DR = 45%	Retrospective	H&N
Yoo et al	2018	MRI * CT	73	DR MR 3D THRIVE = 59% * DR MRI Spin Echo = 40% * DR CT = 29%	Retrospective	H&N
Wolpert et al	2018	CT	189	DR = 58%	Retrospective	Brain
Upadhya et al	2019	CT	53	DR = 43%	Prospective	H&N
Rimer et al	2023	CT	193	DR = 18%	Retrospective	Mixed
Choi et al	2023	MRI	32	DR = 25%	Retrospective	Breast

#### Computed tomography

The first published results on the use of CT scans in CUP came from McMillan et al in 1982. In their cohort of 46 patients, CT identified the primary tumour site in 16 patients (34.8%), compared to only four (8.7%) determined by conventional imaging modalities.^40^ Within the same year, another study at the Mayo Clinic supported these findings and reported a similar 32% detection rate.^41^ Beyond improved primary tumour localization, it was also shown that CT revealed a significant number of previously undetected metastases.^40^^,^^41^ Early research on CT also showcased the added value of CT in head and neck CUP, a clinical scenario previously less accessible with older imaging methods.^42^^,^^43^

The expanding body of evidence supporting CT’s application across diverse clinical contexts, coupled with the significant 3-4-fold improvement demonstrated over conventional roentgenographic modalities in locating primary tumours in CUP, helped establish CT as the core imaging modality of standard CUP workups. CT became the benchmark against which all subsequent imaging modalities for CUP would be compared to validate their effectiveness and added value.

#### Magnetic resonance imaging

The integration of MRI into the CUP clinical workflow has focused on specific body parts and was most extensively studied in breast and head and neck.

Patients with occult breast cancer typically present with isolated axillary lymphadenopathy without any indication of a primary breast tumour upon physical examination and mammography. Using MRI, evidence indicates 70%-100% primary breast tumour detection rates, establishing breast MRI as a cornerstone in the standard CUP workup for these patients.^44–50^ In two separate small-sized studies by Tilanus-Linthorst et al and Henry-Tillman et al, MRI accurately identified or ruled out the existence of a primary breast tumour across all the study participants, while baseline diagnostics, including mammography and ultrasound, were inconclusive.^44^^,^^45^ These results were partially supported by larger studies reporting 70%-86% detection rates.^46–50^ A limited number of studies where breast MRI had lower performance (DR < 70%) still report a notable 25%-50% improvement in detection rates compared to mammography.^51–54^ The addition of breast MRI has also been shown to impact therapeutic management. Prior to 1980, axillary dissection and mastectomy were the standard-of-care for patients with suspected occult breast cancer. Even patients with negative scans were referred for mastectomy; an approach supported by 80%-100% identification of a primary tumour in the breast at histopathology despite negative mammography findings. MRI has drastically reduced the likelihood of detecting a breast tumour after a negative scan, thereby diminishing the necessity for routine (and futile) mastectomies.^44^ So far, it has not been demonstrated that the improved detection rate of breast MRI also impacts survival; Choi et al report comparable outcomes between those with primary tumour identified and those whose primary site remained obscured but had received the standard treatment.^55^

With respect to H&N tumours, the higher image resolution and better tissue characterization of MRI make it a suitable choice for examining patients with cervical metastases of unknown primary. Though the overall body of evidence is smaller than for breast MRI, there have been several reports on the value of MRI in this setting. Gődény et al retrospectively studied a cohort of 38 patients and showed a 16% primary tumour detection rate in patients with cervical lymph node metastases with conventional native T1-, T2-weighted, and STIR sequences. Detection rates increased to 37% when native sequences were combined with DWI, and reached 40% using fat suppression contrast-enhanced T1-weighted measurement and multiparametric MRI.^56^ The significance of selecting task-appropriate MRI sequences is further emphasized by another study from Yoo et al who showed that contrast-enhanced 3D-THRIVE MRI sequences exhibited a 19% higher detection rate compared to conventional spin-echo sequences in H&N cancers of unknown primary.^57^

Evidence on the use of dedicated MRI protocols in other body parts to aid in detecting the primary tumour in CUP is limited. For example, He et al applied pelvic MRI to differentiate primary cervical from endometrial cancer in cases with inconclusive biopsy,^58^ and Al Ansari et al showed that specific characteristics on liver MRI may aid in differentiation between mass-forming cholangiocarcinoma and liver metastases from unknown primary.^59^

#### Summary of pearls and pitfalls of 3D radiologic imaging

CT remains the cornerstone of CUP imaging with the initial diagnostic workup of CUP patients typically including a contrast-enhanced CT scan. This modality is adept at identifying morphologically distinct tumours but is often augmented by other imaging techniques (e.g., FDG-PET/CT and MRI) to overcome its shortcomings in specific clinical contexts, such as breast, abdominopelvic, and head and neck malignancies. MRI offers higher soft tissue contrast and is particularly valuable for detecting and characterizing small lesions in complex anatomical regions, thereby enhancing the diagnostic workflow for CUP patients and impacting patient management. However, there as well, the limitations of MR as a modality need to be considered, especially with the prevalence of lesions of unknown primary in the lungs.

### FDG-PET/CT

PET, specifically FDG-PET, remains the most extensively researched imaging modality in CUP imaging literature (71% of the 140 articles included in this review, Figure 2B). FDG-PET(/CT) CUP literature has examined several endpoints including primary tumour detection, delineating the disease extent, and impact on treatment plans and outcomes. An overview of available literature for FDG-PET and PET/CT for primary tumour detection is provided in Tables 3-5.

**Table 3.** Summary of included studies that reported primary tumour detection rates using FDG-PET(/CT) in cervical metastases of unknown origin.

Authors	Year	Modality	Study Population	Primary tumour detection rate	Study type
Braams et al	1997	18F-FDG PET	13	DR = 23%	Prospective
Stokkel et al	1999	18F-FDG PET	10	DR = 50%	Prospective
AAssar et al	1999	18F-FDG PET	17	DR = 53%	Retrospective
Greven et al	1999	18F-FDG PET	13	DR = 8%	Prospective
Talbot et al	2000	18F-FDG PET	4	DR = 75%	Prospective
Jungehülsing et al	2000	18F-FDG PET	27	DR = 26%	Prospective
Regelink et al	2002	18F-FDG PET	50	DR = 32%	Retrospective
Johansen et al	2002	18F-FDG PET	42	DR = 24%	Prospective
Wong et al	2003	18F-FDG PET	17	DR = 47%	Retrospective
Stoeckli et al	2003	18F-FDG PET	18	DR = 28%	Prospective
Fogarty et al	2003	18F-FDG PET	21	DR = 5%	Retrospective
Miller et al	2005	18F-FDG PET	26	DR = 31%	Prospective
Freudenberg et al	2005	18F-FDG PET/CT * 18F-FDG PET	21	DR = 57% * DR = 52%	Retrospective
Wartski et al	2007	18F-FDG PET/CT	38	DR = 34%	Retrospective
Paul et al	2007	18F-FDG PET(/CT)	14	DR = 50%	Retrospective
Nassenstein et al	2007	18F-FDG PET/CT * 18F-FDG PET	39	DR = 28% * DR = 26%	Prospective
Johansen et al	2008	18F-FDG PET	60	DR = 30%	Prospective
Waltonen et al	2009	18F-FDG PET * 18F-FDG PET/CT	183	DR = 15% * DR = 44%	Retrospective
Roh et al	2009	18F-FDG PET/CT	44	DR = 32%	Prospective
Padovani et al	2009	18F-FDG PET	13	DR = 54%	Prospective
Hu et al	2009	18F-FDG PET/CT	93	DR = 43%	Retrospective
Yabuki et al	2010	18F-FDG PET	24	DR = 37.5%	Retrospective
Rudmik et al	2011	18F-FDG PET/CT	20	DR = 55%	Prospective
Keller et al	2011	18F-FDG PET/CT * 18F-FDG PET	77	DR = 37% * DR = 15%	Retrospective
Deron et al	2011	18F-FDG PET/CT	18	DR = 0%	Prospective
Dandekar et al	2011	18F-FDG PET/CT	112	DR = 22%	Retrospective
Zhao et al	2012	18F-FDG PET/CT	25	DR = 44%	Retrospective
Pereira et al	2012	18F-FDG PET	49	DR = 18%	Retrospective
Karapolat et al	2012	18F-FDG PET/CT	20	DR = 35%	Retrospective
Chen et al	2012	18F-FDG PET/CT	27	DR = 41%	Retrospective
Prowse et al	2013	18F-FDG PET/CT	32	DR = 50%	Retrospective
Lee et al	2013	18F-FDG PET/CT	37	DR = 38%	Retrospective
Majchrzak et al	2015	18F-FDG PET/CT	41	DR = 17%	Retrospective
Lee et al	2015	18F-FDG PET/CT	56	DR = 38%	Prospective
Su et al	2016	18F-FDG PET/CT	54	DR = 24%	Retrospective
Mani et al	2016	18F-FDG PET/CT	52	DR = 46%	Retrospective
Godeny et al	2016	18F-FDG PET/CT	38	DR = 45%	Retrospective
Dale et al	2017	18F-FDG PET/CT	30	DR = 3%	Retrospective
Liu et al	2019	18F-FDG PET/CT	40	DR = 40%	Retrospective
Sarma et al	2021	18F-FDG PET/CT	63	DR = 29%	Retrospective
Chen et al	2021	18F-FDG PET/CT	37	DR = 41%	Retrospective
Kanodia et al	2022	18F-FDG PET/CT	22	DR = 14%	Prospective
Eilsberger et al	2022	18F-FDG PET/CT	31	DR = 39%	Retrospective

**Table 4.** Summary of included studies that reported primary tumour detection rates using FDG-PET(/CT) in bone and brain metastases of unknown origin.

Authors	Year	Modality	Study population	Primary tumour detection rate	Study type	Specific anatomical region
Klee et al	2002	18F-FDG PET	16	DR = 50%	Retrospective	Brain
Tamam et al	2016	18F-FDG PET/CT	87	DR = 74%	Retrospective	Bone
Wolpert et al	2018	18F-FDG PET/CT	189	DR = 20%	Retrospective	Brain
Koç et al	2018	18F-FDG PET/CT	26	DR = 77%	Retrospective	Brain
Lawrenz et al	2020	18F-FDG PET/CT	13	DR = 8%	Retrospective	Bone
Budak et al	2020	18F-FDG PET/CT	100	DR = 72%	Retrospective	Bone
Mohamed et al	2021	18F-FDG PET/CT	39	DR = 49%	Prospective	Brain

**Table 5.** Summary of included studies that reported primary tumour detection rates using FDG-PET(/CT) in patient cohorts with metastases of unknown origin (either with mixed lesion locations or with specific organs other than the neck, brain, and bones).

Authors	Year	Modality	Study population	Primary tumour detection rate	Study type	Specific anatomical region
Kole et al	1998	18F-FDG PET	29	DR total = 24% * DR cervical = 25% * DR extracervical = 23%	Prospective	Mixed
Lassen et al	1999	18F-FDG PET	20	DR = 45%	Prospective	Mixed
Trampal et al	2000	18F-FDG PET	12	DR = 33%	Prospective	Mixed
Lonneux et al	2000	18F-FDG PET	24	DR = 54%	Retrospective	Mixed
Bohuslavizki et al	2000	18F-FDG PET	53	DR = 38%	Retrospective	Mixed
Rades et al	2001	18F-FDG PET	42	DR = 43%	Prospective	Mixed
Zhao et al	2003	18F-FDG PET	50	DR = 64% * DR cervical = 78% * DR bone = 67% * DR brain = 42%	Retrospective	Mixed
Mantaka et al	2003	18F-FDG PET	25	DR = 48%	Prospective	Mixed
Alberini et al	2003	18F-FDG PET	41	DR = 63%	Retrospective	Mixed
Joshi et al	2004	18F-FDG PET	62	DR = 26%	Retrospective	Extracervical
Scott et al	2005	18F-FDG PET	31	DR = 26%	Retrospective	Extracervical
Nanni et al	2005	18F-FDG PET/CT	21	DR = 57%	Prospective	Mixed
Kolesnikov-Gauthier et al	2005	18F-FDG PET	24	DR = 25%	Prospective	Mixed
Gutzeit et al	2005	18F-FDG PET/CT * 18F-FDG PET	45	DR = 33% * DR = 24%	Retrospective	Mixed
Pelosi et al	2006	18F-FDG PET/CT	68	DR = 35%	Retrospective	Mixed
Garin et al	2007	18F-FDG PET	51	DR = 24%	Prospective	Mixed
Fencl et al	2007	18F-FDG PET/CT	190	DR = 16%	Retrospective	Mixed
Kaya et al	2008	18F-FDG PET/CT	43	DR = 56%	Retrospective	Mixed
Park et al	2011	18F-FDG PET(/CT)	20	DR = 0%	Retrospective	Mixed
Pak et al	2011	18F-FDG PET/CT	51	DR = 10%	Retrospective	Mixed
Hu et al	2011	18F-FDG PET/CT	149	DR = 25%	Retrospective	Mixed
Tamam et al	2012	18F-FDG PET/CT	316	DR = 75%	Retrospective	Mixed
Møller et al	2012	18F-FDG PET/CT	135	DR = 28%	Prospective	Extracervical
Han et al	2012	18F-FDG PET/CT	120	DR = 43%	Retrospective	Mixed
Saidha et al	2013	18F-FDG PET/CT	50	DR = 50% * DR cervical = 69% * DR extracervical = 41%	Retrospective	Mixed
Deonarine et al	2013	18F-FDG PET/CT	51	DR = 37%	Retrospective	Mixed
Sürücüa et al	2014	18F-FDG PET/CT	58	DR = 65%	Retrospective	Mixed
Elboga et al	2014	18F-FDG PET/CT	112	DR = 33%	Retrospective	Mixed
Breuer et al	2014	18F-FDG PET/CT	70	DR = 26%	Retrospective	Mixed
Yu et al	2016	18F-FDG PET/CT	449	DR = 26%	Retrospective	Mixed
Yaylali et al	2016	18F-FDG PET/CT	32	DR = 50%	Retrospective	Mixed
Riaz et al	2016	18F-FDG PET/CT	82	DR = 57%	Retrospective	Mixed
Wafaie et al	2018	18F-FDG PET/CT	52	DR = 56%	Prospective	Mixed
Thapa et al	2018	18F-FDG PET/CT	21	DR = 57%	Retrospective	Mixed
Cetin Avci et al	2018	18F-FDG PET/CT	36	DR = 67%	Retrospective	Mixed
Cengiz et al	2018	18F-FDG PET/CT	121	DR = 49%	Retrospective	Mixed
Zytoon et al	2020	18F-FDG PET/CT	175	DR = 57%	Prospective	Mixed
Li et al	2020	18F-FDG PET/CT	124	DR = 77%	Retrospective	Liver
Fatima et al	2020	18F-FDG PET/CT	46	DR = 57%	Prospective	Mixed
Soni et al	2021	18F-FDG PET/CT	83	DR = 39%	Retrospective	Extracervical
Nissan et al	2021	18F-FDG PET/CT	64	DR = 44%	Retrospective	Mixed
Nikolova et al	2021	18F-FDG PET/CT	53	DR = 36%	Retrospective	Lymph nodes
Bicakci et al	2022	18F-FDG PET/CT	155	DR = 41%	Retrospective	Mixed
Atilgan et al	2022	18F-FDG PET/CT	68	DR = 76%	Retrospective	Mixed
Ahmad et al	2023	18F-FDG PET/CT	187	DR = 50%	Retrospective	Mixed
Rimer et al	2023	18F-FDG PET/CT	193	DR = 36.5%	Retrospective	Mixed
Huang et al	2024	18F-FDG PET/CT	62	DR = 68%	Retrospective	Mixed

#### Primary tumour detection

The reported efficacy of FDG-PET alone in detecting the primary tumours in CUP varies significantly, with detection rates ranging from as low as 5% to as high as 75%.^60–74^

Early studies combining FDG-PET with CT demonstrated improved performance, reporting detection rates of 33%-57%.^75–78^ Direct comparisons confirmed that PET/CT outperforms PET alone, achieving DRs of 28%-57% compared to 15%-52%, respectively.^77–81^ Subsequent studies further corroborated these findings, highlighting a consistent improvement in diagnostic accuracy with PET/CT.^82–94^

Burglin et al in a pooled analysis of 20 studies, including 1942 CUP patients, reported a pooled detection rate of approximately 41% for PET/CT to establish a primary tumour diagnosis.^95^ A more recent individual patient data meta-analysis by Willemse et al, including 1865 patients, showed that DRs for PET vary depending on the dominant pattern of metastatic spread with highest pooled DRs observed in patients presenting with brain metastases (DR = 74%) or liver metastases (DR = 54%) compared to low DRs in patients with peritoneal or lymph node dominant disease (DRs = 37%-38%).^96^

Other specific subgroups that have received particular attention in literature are highlighted below:

##### Cervical lymph node metastases of unknown primary

Numerous FDG-PET(/CT) studies have focused on this specific type of CUP since the soft tissue of the neck is not visualized well using contrast-enhanced CT. The sensitivity of FDG-PET/CT in detecting primary tumours in cervical CUP has been extensively documented to be higher than that of conventional imaging methods.^97^ Moreover, detection rates of FDG-PET/CT for cervical CUP (DR = 61%) have been shown superior to those in CUP patients with extracervical disease (DR = 40%).^98^ Other reported detection rates for FDG-PET(/CT) in cervical CUP ranged from 7% to 84.6% across various studies^70^^,^^77^^,^^80^^,^^83^^,^^99–117^ (Table 3).

##### Bone metastases of unknown primary

Bone metastases of unknown primary, though less common than other conditions, present a substantial diagnostic challenge.^118^ The role of FDG-PET/CT in diagnosing bone CUP is highlighted by several studies (Table 4), including one of the largest in CUP imaging research, conducted by Yu et al. This study, which involved 449 CUP patients, reported an overall detection rate of 25.6% using PET/CT. While observing comparable performance across different anatomical regions, they achieved the highest DR within the subgroup of bone CUP: head and neck (29.1%), bone (38.8%), and cerebrum (22.7%).^119^ The authors strongly advocated for the early use of PET/CT in diagnostic evaluation of these patients, and emphasized that its application, where routine examinations are inconclusive, may still benefit certain patient groups. In a pooled meta-analysis of FDG-PET/CT studies in CUP, a detection rate of 49% was reported for bone-predominant metastases, with lung and prostate cancers emerging as the most common primary tumours.^96^ Notably, some studies have reported primary tumour detection rates exceeding 70% in bone CUP; possibly due to less thorough initial diagnostic workups, a common confounding factor in CUP research that should always be considered while interpreting the findings.^120^^,^^121^

##### Brain metastases of unknown primary

The efficacy of FDG-PET/CT in detecting primary tumours in brain CUP shows mixed results across different studies (Table 4). Wolpert et al found that FDG-PET/CT was not superior to chest/abdomen CT in localizing the primary lesion.^122^ This finding is likely influenced by the predominance of lung cancers in their cohort, which CT is known to identify effectively. Conversely, some studies report higher detection rates in line with other presentations of CUP.^123–125^ In particular, Koç et al report a 77% detection rate in their cohort of 26 patients with presumed brain metastases of unknown primary, though their initial diagnostic workup involved limited imaging.^124^ A pooled meta-analysis demonstrated that FDG-PET/CT achieves the highest detection rate of 74% in CUPs with brain-dominant presentations, likely reflecting the high prevalence of hypermetabolic lung and brain primary tumours in these patients.^96^ While these studies have relatively small patient groups, larger-scale research might help shed light on the optimal role of PET/CT in this patient population.

#### Detecting the extent of metastatic spread

FDG-PET/CT has shown favourable sensitivity for determining the extent of metastatic disease (94%), with better results compared to its ability to detect primary lesions (sensitivity = 62%).^126^ Compared to conventional imaging (mainly CT), PET/CT is generally superior for detecting additional metastatic sites.^62^^,^^127–129^ CUP imaging studies have reported varied results in identifying undetected tumour sites using PET/CT, with detection rates ranging from 28.5% to 54.9%.^60^^,^^64^^,^^82^^,^^91^^,^^100^^,^^130–134^ Studies have demonstrated that PET/CT is particularly effective for identifying lesions in the brain, bone, and liver, where its sensitivity remains robust.^96^ For less metabolically active tumour types such as mucinous carcinomas, PET/CT will be less sensitive. Furthermore, PET/CT may in particular be advantageous in patients with widespread or atypical metastatic patterns that are more difficult to detect on conventional imaging.

#### Impact on therapeutic decision-making and clinical outcomes

Literature reports significant influence of PET(/CT) on therapeutic decision-making in CUP patients, ranging from 24% to 69% of cases.^64^^,^^75^^,^^82^^,^^83^^,^^130^^,^^135–140^ These modifications are normally prompted by the detection of previously unknown lesions, or by the identification of the primary tumour. For instance, in Johansen et al’s study including 42 cervical CUP patients, the additional information obtained from FDG-PET/CT resulted in reducing the irradiation fields in six, preventing unhelpful radical treatments in two, adding surgical procedures in one, and using accelerated radiotherapy in another patient.^135^

Moreover, the ability of PET/CT to accurately delineate the disease extent is particularly relevant as CUP outcomes vary based on locoregional or systemic involvement. For example, patients with cervical CUP have been documented to achieve better survival rates than those with extracervical CUP.^137^ Breuer et al corroborated this by revealing notable differences in 1-year survival rates of CUP patients: 78% in those with locoregional and 34% in patients with disseminated disease.^141^ Additionally, the number of metastatic lesions and the degree of dissemination are independently associated with survival.^91^^,^^142^ Soni et al observed that CUP patients with oligometastatic disease (<3 lesions) had a significantly better median survival (2.16 years) than those with disseminated disease (0.67 years).^85^

However, the survival benefit of primary tumour identification remains contentious. Interestingly, several studies have indicated that discovering the origin does not necessarily translate to improved survival.^61^^,^^91^^,^^137^^,^^141^^,^^143^ For example, Soni et al showed that patients without a subsequent primary diagnosis fared better (median survival = 1 year) than those with an identified primary (median survival = 0.67 years).^85^ Similarly, a cohort of cervical CUP patients demonstrated a 5-year overall survival rate of 52% for those with an unknown primary compared to 22% for those with a detected primary, further questioning the survival advantages of identifying the primary tumour.^106^ Overall, this paradox is symbolic of the complexity of CUP; future research should focus not only on the detection of primary tumours, but also on the overall impact such findings might have on patient management and survival.

#### Summary of pearls and pitfalls of FDG-PET and PET/CT

FDG-PET is a metabolic imaging technique, and its uptake values reflect glycolysis levels. This unique mechanism of action is complementary to conventional imaging approaches, and adds value by uncovering metabolically active lesions which might be morphologically occult. As a result, PET/CT is adept at providing a more comprehensive representation of the true extent of the disease, particularly impactful in patients with FDG-avid tumours where it can modify the therapeutic strategies in up to 69% of CUP patients by detecting new lesions or the primary tumour.

Despite reports showing favourable DRs for PET to discern the primary tumour, some studies have shown contrastingly low detection rates (ranging from 0% to 17%) using PET(/CT).^144–148^ FDG-PET's limitations in detecting primary tumours can stem from different factors. While PET’s unique ability allows for the determination of metastatic spread, standardized uptake values (SUV) may not differ significantly between primary and non-primary lesions of the same patient.^60^^,^^85^^,^^108^

Additionally, while PET(/CT) is a highly sensitive method, it can suffer from lower specificity, leading to false positive results.^136^^,^^149^ False positive rates ranging from 21% to 46% were observed in various studies.^62^^,^^65^^,^^99^^,^^108^ High physiological uptake in regions such as the oropharyngeal area, where many CUPs originate, among other factors can lead to false positive findings.^150^ PET(/CT) can also yield false negative results, particularly in cases with non-FDG-avid tumour types, small tumour size, or primary tumours located in areas with high physiological uptake, masking their presence.^151–154^

Given the points above, there is a debate on where PET(/CT) would best belong in the CUP diagnostic/therapeutic workflow. While PET has value in detecting unknown primary tumours and additional metastatic sites, it is generally considered complementary to other diagnostic modalities according to most clinical guidelines.^3^^,^^155^^,^^156^ Some studies have proposed using PET/CT as a first-line imaging modality to validate the overall tumour spread and better characterize the disease.^157^^,^^158^ Further research is needed to establish the most effective/optimized integration of FDG-PET(/CT) into the diagnostic and therapeutic workflow for CUP patients.

### Emerging technologies

Looking ahead, novel imaging techniques are poised to significantly enhance diagnostic precision and therapeutic guidance in the imaging of CUP.

#### Fibroblast activation protein inhibitor-positron emission tomography

FAPI-PET, which uses a radiolabeled fibroblast activation protein inhibitor (FAPI) as a tracer, represents an innovative molecular imaging approach. FAPI targets fibroblast activation proteins, a group of glycoproteins overexpressed in the stroma of many epithelial cancers.^159^ This targeting mechanism exploits the differential expression of fibroblast activation proteins between cancerous and normal tissues, enhancing tumour-to-background contrast. Compared to traditional FDG-PET/CT, which relies on glucose metabolism for tumour imaging, FAPI-PET offers a potentially more sensitive tumour detection method by visualizing the stromal component of tumours.^160^^,^^161^

As an emerging modality, literature on the complementary role of FAPI-PET in CUP imaging is limited. However, two studies have shown promising results, specifically in head and neck CUP. Gu et al assessed the added value of 68Ga-FAPI PET/CT in detecting primary tumours in patients with H&N CUP, who had negative FDG-PET/CT results.^162^ The study enrolled 18 patients, and 68Ga-FAPI PET/CT successfully identified the primary tumour in 39% (7/18) of the cases. The identified tumours displayed moderate to intense uptake of 68Ga-FAPI, with primary locations including the nasopharynx, palatine tonsil, submandibular gland, and hypopharynx. In a follow-up prospective comparative trial, 68Ga-FAPI PET/CT was compared directly with FDG-PET/CT in a head and neck CUP cohort of 91 patients.^163^ 68Ga-FAPI PET/CT was significantly more effective (DR = 51%) in identifying primary tumours than FDG-PET/CT (DR = 19%). Shu et al also reported a 68% detection rate using 68Ga-FAPI PET/CT following negative or equivocal results with FDG-PET/CT.^164^ Although preliminary, these studies show the potential of FAPI-PET/CT to enhance the diagnostic workflow for at least head and neck cancers of unknown primary.

#### Whole-body MRI and novel molecular imaging

Locoregional MRI is standard-of-care in the workup of CUP to provide detailed images of suspected areas such as the breast, head & neck, brain, and pelvis.^3^ Multiparametric anatomical and functional MRI has enhanced the ability to detect and characterize even small-sized lesions.^165^^,^^166^ The adoption of diffusion-weighted MRI, for instance, has been tested in a wide variety of oncological settings, offering advantages in identifying cancer lesions in various anatomic regions such as the brain, liver, and pancreas.^17^^,^^167^ As whole-body MRI (WB-MRI) gains broader clinical acceptance and popularity, its capacity for state-of-the-art MR imaging across larger body areas might become useful also in the context of CUP with a few initial reports having shown promising results.^168–171^ For example a recent pilot study by Willemse et al showcased the potential benefits of body MRI for CUP in a small study of 30 patients with suspected abdominal malignancies (based on histology and/or pattern of the disease) in whom the routine diagnostic workup including CT and FDG-PET/CT was not able to detect the underlying primary tumour. In specific settings, WB-MRI with DWI could correctly identify a primary tumour in 30% and detect additional metastatic sites in 17% of the patients.^172^ Similarly, Kang et al reported that whole-body DWI was able to detect the primary tumour in a case of cervical metastases of unknown origin, where routine imaging was inconclusive.^173^ Additional research is required to determine whether WB-MRI could truly benefit CUP patients.

Combining the soft tissue resolution of MRI with the molecular imaging of PET has been a long-standing objective of imaging research. However, the use of integrated FDG-PET/MRI in CUP remains underexplored, with limited studies yielding mixed results on its added value for identifying occult primary tumours. Our review identified only three small-scale studies comparing PET/CT with PET/MRI in CUP. Ruhlmann et al reported an equal detection rate of 55% with both modalities, while the other two studies favoured PET/MRI, demonstrating 8% and 32% improvements in detection rates using this modality.^174–176^

Though still in preliminary stages of research, molecular MRI techniques such as 19F MRI, as well as novel PET tracers targeting specific microenvironmental elements (e.g., PD-L1, CD8, LAG-3, etc) are emerging on the horizon. These modalities, if developed into clinical application, might also help unravel the biological complexities of CUP.^177–181^

#### Radiomics and artificial intelligence

Radiomic features, quantifying the shape, texture, and intensity of a region of interest, provide detailed insights into tumours’ (morphological) phenotypes, beyond what is visible to the naked eye.^182^ Studies have linked the quantitative morphological phenotype of tumours to several clinical endpoints such as therapeutic response, prognosis, and tumour biology.^183–190^

While more AI/radiomic studies have yet to be published to address the needs of CUP, two studies have harnessed radiomics to look at morphological differences between different tumour types. In a small monocentric study, MRI texture analysis was used to differentiate brain metastases' primary site of origin with remarkable results, especially in discriminating between lung cancer from breast and melanoma.^191^ Similarly, Tomita et al identified morphological characteristics specific to cervical lymph nodes originating from lymphomas.^192^ While these studies did not explore radiomics in CUP cohorts, their results on routine diagnostic patient populations signal a potential for future translation. The only radiomic study directly working with CUP patients was Ishiwata et al, where features extracted from FDG-PET/CT images (*n* = 30 patients) predicted overall survival.^193^

Beyond radiological imaging, artificial intelligence has recently been applied to other clinically relevant data to identify the primary tumour sites in CUP. These models have been trained using histopathology, cytology, next-generation sequencing, or whole genome sequencing data.^194–197^ As AI methods evolve, they hold great potential to improve the precision and efficiency of diagnostic workups in CUP.

### Clinical diagnostic workflows and multimodality imaging in CUP

During the initial suspicion of potential malignancy, patients are usually subject to a rapid, systematic screening process that begins with widely available, low-cost imaging modalities. 2D tools such as X-ray and ultrasound are often employed first due to their accessibility. Contrast-enhanced CT, the backbone of oncologic imaging, is subsequently integrated to provide detailed cross-sectional anatomical information, (full) body coverage to assess potential metastatic spread, and better characterize equivocal findings.

When this conventional workup fails to identify a primary tumour, patients may be categorized using terms such as “provisional CUP”, “unconfirmed CUP”, or “malignancy of undefined origin (MUO)” according to the NICE guidelines.^1^ ESMO guidelines recommend that these patients are further evaluated using (if not yet acquired) contrast-enhanced CT or MRI of the neck, chest, abdomen, and pelvis, supplemented by mammography for female patients, as the minimum standard. Additional imaging including MRI and/or PET/CT may then be tailored to specific clinical scenarios, such as suspected head and neck primary, salivary gland carcinoma, or breast cancer.^3^

If the diagnostic workup remains inconclusive, the diagnosis of CUP is established. Imaging at this stage plays a critical role in guiding treatment and monitoring the response. For patients with single-lesion or oligometastatic CUP, whole-body FDG-PET/CT and brain MRI should be performed according to ESMO guidelines to rule out further metastases before considering radical locoregional treatment. Long-term management includes follow-up imaging every 3-6 months, using CT or MRI, to assess treatment outcomes and identify potential late-emerging primary tumours.

Building on these established imaging protocols, recent investigations have focused on refining the diagnostic processes further by combining advanced imaging modalities and interventions to address the inherent limitations of individual techniques. Combining FDG-PET with other diagnostic methods has been suggested to mitigate shortcomings in primary tumour detection. Regelink et al demonstrated that combining FDG-PET, CT/MR, and panendoscopy identified primary tumours in 16 patients with cervical CUP (DR = 32%), with four primaries uncovered exclusively by FDG-PET.^198^ Furthermore, using PET/CT alongside endoscopy under anaesthesia has been reported to increase the negative predictive value to 100% when both tests are negative and the positive predictive value to 100% when both tests are positive.^199^ Willemse et al showed that the addition of body MRI following PET/CT allowed for the localization of several primary tumour sites that were detected but misclassified on PET/CT, highlighting the complementary value of the two techniques.^172^ Based on the clinical scenario, it might be possible to combine multiple modalities to best improve their diagnostic accuracy.

Looking ahead, the evolution of CUP diagnostics will likely hinge on integrating imaging findings with molecular data. Recent studies demonstrate that comprehensive gene expression profiling can identify the likely tissue of origin in a substantial proportion of CUP patients.^196^ Whole genome sequencing and other molecular analyses can yield insights into tumour biology, potentially guiding targeted therapies and helping to pinpoint the tissue of origin when imaging alone is inconclusive. This new paradigm, where imaging and genomics inform one another, may refine diagnostic pathways and improve patient stratification.

Building on these insights, it may be valuable to consider the implementation of dedicated multidisciplinary CUP teams, composed at least of an oncologist, radiologist, and pathologist, as a standard feature in the clinical settings.^1^ Such teams could streamline diagnostic pathways, reduce delays, and ultimately improve both diagnostic yield and patient experience. Future efforts should focus on systematically evaluating how these strategies influence workflow efficiency, treatment outcomes, and overall satisfaction among patients and healthcare professionals. Such studies can help guide the development of evidence-based guidelines and provide patients and their caregivers with greater clarity in the face of this challenging diagnosis.

## Conclusion

Early medical imaging techniques, despite their notable limitations, enabled the routine identification of primary tumours, with cases that remained undiagnosed, being classified as CUP. Advances in modern imaging modalities such as CT, MRI, and PET/CT have significantly overcome these limitations, improving primary tumour detection rates. Particularly, contrast-enhanced CT and MRI have increased tumour localization and characterization, with MRI being helpful in cases of occult breast, head and neck, and abdominopelvic cancers. FDG-PET/CT, integrating metabolic and anatomical imaging, has added value in assessing metastatic spread and primary tumour detection, leading to meaningful modifications in treatment plans, despite the challenges with false positives and negatives. Emerging technologies like FAPI-PET/CT, AI/radiomics, and whole-body MRI hold promise for further advancements.

However, CUP imaging research continues to grapple with a range of critical unresolved questions and challenges, spanning both foundational concepts and practical applications. It remains unclear whether a primary tumour actually exists in all cases or if it might be absent for various reasons (i.e., “false” vs “true” CUP). Additionally, it is uncertain whether identifying the primary tumour consistently improves outcomes or offers other benefits. Practically, the field struggles with challenges in distinguishing primary tumours from metastatic lesions, determining the ideal combination and sequence of imaging modalities in diagnostic workups, and exploring where to draw the line of confirming CUP as the final diagnosis.

Moving forward, research must address these gaps, though the reliability of its findings will first need to be strengthened by resolving the inherent biases in CUP research. These biases typically arise from variations in how CUP is understood across different studies and institutions. This often results in considerable heterogeneity in patient selection, the quantity and quality of baseline diagnostics, and ultimately, the reported outcomes, to the extent that studies become too dissimilar to be aggregated for reliable conclusions.

To improve the interpretability and applicability of research findings, multi-center prospective studies with consistent inclusion criteria are essential. Retrospective studies should also consider re-evaluating patients’ eligibility, as evidence suggests that over 30% of CUP cases in registries could actually be reclassified as known primaries.^200^ Multi-center collaborations between established CUP centres should be set up with multiple clear objectives: overcoming data scarcity, harmonizing the differences in tumour type distribution, and dissemination of experience between multidisciplinary tumour board members. Notwithstanding the inherent ambiguity in the diagnostic label of CUP, such large-scale consortium initiatives might allow for greater insights into this disease and how best to manage it.

## Supplementary Material

tqaf039_Supplementary_Data
