# Supplementary material for: Imaging of cancer of unknown primary: a systematic literature review of the past, present, and future
Source: Br J Radiol. 2025 Mar 21;98(1172):1209–26. doi: 10.1093/bjr/tqaf039 (PMC12341689; doi:10.1093/bjr/tqaf039)
Supplement: tqaf039_Supplementary_Data [file tqaf039_supplementary_data.zip › tqaf039_Supplementary_Data/CUP_Revision_Supplementary_CLEAN.docx]

**Supplementary Material**

**Supplementary Table 1. Description of the search strategy**

**MEDLINE**

|  | **Search term** | **n_studies** |
| --- | --- | --- |
| 1 | Neoplasms, Unknown Primary/ | 4064 |
| 2 | ((Occult or unknown or undefined or hidden or obscure or discern) adj3 primary adj6 (cancer* or neoplas* or carcinoma* or tumor* or tumor* or malignan*)).ti,ab,kf. | 4346 |
| 3 | ((muo or cup) and (cancer* or neoplas* or carcinoma* or tumor* or tumour* or malignan*)).ti,ab,kf. | 2141 |
| 4 | ("malignanc* of unknown origin" or "cancer* of unknown origin" or "neoplas* of unknown origin" or "carcinoma* of unknown origin" or "tumor* of unknown origin" or "tumor* of unknown origin").ti,ab,kf. | 698 |
| 5 | 1 or 2 or 3 or 4 | 8186 |
| 6 | exp diagnostic imaging/ | 2984617 |
| 7 | (imaging* or "computer-assisted image interpretation" or "radiographic-assisted image interpretation").ti,ab,kf. | 1139859 |
| 8 | exp Tomography, X-Ray Computed/ | 507352 |
| 9 | ("computed tomograph*" or CT or "computer assisted tomograph*" or "computerized tomograph*" or "CAT scan*" or "electron beam tomograph*" or "computerized axial tomograph*" or spect*).ti,ab,kf. | 2304757 |
| 10 | exp Magnetic Resonance Imaging/ | 553817 |
| 11 | ("magnetic resonance imag*" or "nmr imag*" or "mr tomograph*" or mri or mris or fmri* or "nmr tomograph*" or zeugmatograph* or "chemical shift imaging*" or "magnetization transfer contrast imaging*").ti,ab,kf. | 550555 |
| 12 | exp Positron-Emission Tomography/ | 83762 |
| 13 | ("positron emission tomograph*" or "petscan*" or pet).ti,ab,kf. | 162088 |
| 14 | (echocardiograp* or holograph* or "microwave tomograph*" or "cell tracking*" or neuroimaging* or "brain cortical thickness" or "brain mapping" or neuroradiograph* or "cerebral angiograph*" or "cerebral ventriculograph*" or echoencephalograph* or myelograph* or pneumoencophalograph* or echoencephalograph* or myelograph* or photography or photographies or holography or holographies or photofluorograph* or photogrammetr* or "moire topograph*" or "radiostereometric analys*" or radiograph* or "photon absorptiometry" or x-ray* or xray* or "dxa scan*" or "dexa scan*" or angiograph* or arthrograph* or electrokymograph* or fluoroscop* or hysterosalpingograph* or lymphograp* or mammograph* or microradiograph* or neuroradiograph* or pneumoradiograph* or urograph* or xeroradiograph* or "radioisotope scan*" or scintigraph* or scintiphotograph* or lymphoscintigraph* or radioimmunodetection* or "radiolabelled immunoscintigraph*" or radioimmunoimaging* or radioimmunoscintigraph* or "radiolabeled immunoscintigraph*" or "ventilation-perfusion scan*" or "v-q scintigraph*" or "vq lung scan*" or "lung vq scan*" or "near-infrared spectroscop*" or "nir spectroscop*" or "near-infrared spectrometr*" or "nir spectrometr*" or "subtraction technique*" or thermograph* or "temperature mapping*" or echocardiograph* or endosconograph* or ultrasonograph* or "whole body imaging*" or ultrasound* or "whole body scan*" or "whole body screening*").ti,ab,kf. | 1738560 |
| 15 | 6 or 7 or 8 or 9 or 10 or 11 or 12 or 13 or 14 | 5799783 |
| 16 | 5 and 15 | 3035 |

**Embase**

|  | **Query** | **Last results** |
| --- | --- | --- |
| #17 | #16 NOT ('conference abstract'/it OR 'conference paper'/it OR 'conference review'/it) | 3,299 |
| #16 | #5 AND #15 | 4,912 |
| #15 | #6 OR #7 OR #8 OR #9 OR #10 OR #11 OR #12 OR #13 OR #14 | 6,291,458 |
| #14 | echocardiograp*:ti,ab,kw OR holograph*:ti,ab,kw OR 'microwave tomograph*':ti,ab,kw OR 'cell tracking*':ti,ab,kw OR neuroimaging*:ti,ab,kw OR 'brain cortical thickness':ti,ab,kw OR 'brain mapping':ti,ab,kw OR 'cerebral angiograph*':ti,ab,kw OR 'cerebral ventriculograph*':ti,ab,kw OR pneumoencophalograph*:ti,ab,kw OR echoencephalograph*:ti,ab,kw OR myelograph*:ti,ab,kw OR photography:ti,ab,kw OR photographies:ti,ab,kw OR holography:ti,ab,kw OR holographies:ti,ab,kw OR photofluorograph*:ti,ab,kw OR photogrammetr*:ti,ab,kw OR 'moire topograph*':ti,ab,kw OR 'radiostereometric analys*':ti,ab,kw OR radiograph*:ti,ab,kw OR 'photon absorptiometry':ti,ab,kw OR 'x ray*':ti,ab,kw OR xray*:ti,ab,kw OR 'dxa scan*':ti,ab,kw OR 'dexa scan*':ti,ab,kw OR angiograph*:ti,ab,kw OR arthrograph*:ti,ab,kw OR electrokymograph*:ti,ab,kw OR fluoroscop*:ti,ab,kw OR hysterosalpingograph*:ti,ab,kw OR lymphograp*:ti,ab,kw OR mammograph*:ti,ab,kw OR microradiograph*:ti,ab,kw OR neuroradiograph*:ti,ab,kw OR pneumoradiograph*:ti,ab,kw OR urograph*:ti,ab,kw OR xeroradiograph*:ti,ab,kw OR 'radioisotope scan*':ti,ab,kw OR scintigraph*:ti,ab,kw OR scintiphotograph*:ti,ab,kw OR lymphoscintigraph*:ti,ab,kw OR radioimmunodetection*:ti,ab,kw OR 'radiolabelled immunoscintigraph*':ti,ab,kw OR radioimmunoimaging*:ti,ab,kw OR radioimmunoscintigraph*:ti,ab,kw OR 'radiolabeled immunoscintigraph*':ti,ab,kw OR 'ventilation-perfusion scan*':ti,ab,kw OR 'v-q scintigraph*':ti,ab,kw OR 'vq lung scan*':ti,ab,kw OR 'lung vq scan*':ti,ab,kw OR 'near-infrared spectroscop*':ti,ab,kw OR 'nir spectroscop*':ti,ab,kw OR 'near-infrared spectrometr*':ti,ab,kw OR 'nir spectrometr*':ti,ab,kw OR 'subtraction technique*':ti,ab,kw OR thermograph*:ti,ab,kw OR 'temperature mapping*':ti,ab,kw OR echocardiograph*:ti,ab,kw OR endosconograph*:ti,ab,kw OR ultrasonograph*:ti,ab,kw OR 'whole body imaging*':ti,ab,kw OR ultrasound*:ti,ab,kw OR 'whole body scan*':ti,ab,kw OR 'whole body screening*':ti,ab,kw | 2,353,848 |
| #13 | 'positron emission tomograph*':ti,ab,kw OR 'petscan*':ti,ab,kw OR pet:ti,ab,kw | 270,058 |
| #12 | 'positron emission tomography'/exp | 251,500 |
| #11 | 'magnetic resonance imag*':ti,ab,kw OR 'nmr imag*':ti,ab,kw OR 'mr tomograph*':ti,ab,kw OR mri:ti,ab,kw OR mris:ti,ab,kw OR fmri*:ti,ab,kw OR 'nmr tomograph*':ti,ab,kw OR zeugmatograph*:ti,ab,kw OR 'chemical shift imaging*':ti,ab,kw OR 'magnetization transfer contrast imaging*':ti,ab,kw | 855,107 |
| #10 | 'nuclear magnetic resonance imaging'/exp | 1,322,604 |
| #9 | 'computed tomograph*':ti,ab,kw OR ct:ti,ab,kw OR 'computer assisted tomograph*':ti,ab,kw OR 'computerized tomograph*':ti,ab,kw OR 'cat scan*':ti,ab,kw OR 'electron beam tomograph*':ti,ab,kw OR 'computerized axial tomograph*':ti,ab,kw OR spect*:ti,ab,kw | 2,910,614 |
| #8 | 'x-ray computed tomography'/exp | 114,012 |
| #7 | imaging*:ti,ab,kw OR 'computer-assisted image interpretation':ti,ab,kw OR 'radiographic-assisted image interpretation':ti,ab,kw | 1,598,453 |
| #6 | 'diagnostic imaging'/exp | 275,836 |
| #5 | #1 OR #2 OR #3 OR #4 | 12,772 |
| #4 | malignanc* of unknown origin':ti,ab,kw OR 'cancer* of unknown origin':ti,ab,kw OR 'neoplas* of unknown origin':ti,ab,kw OR 'carcinoma* of unknown origin':ti,ab,kw OR 'tumor* of unknown origin':ti,ab,kw OR 'tumour* of unknown origin':ti,ab,kw | 1,048 |
| #3 | (muo:ti,ab,kw OR cup:ti,ab,kw) AND (cancer*:ti,ab,kw OR neoplas*:ti,ab,kw OR carcinoma*:ti,ab,kw OR tumor*:ti,ab,kw OR tumour*:ti,ab,kw OR malignan*:ti,ab,kw) | 3,819 |
| #2 | ((occult OR unknown OR undefined OR hidden OR obscure OR discern) NEAR/3 primary NEAR/6 (cancer* OR neoplas* OR carcinoma* OR tumor* OR tumour* OR malignan*)):ti,ab,kw | 6,992 |
| #1 | 'cancer of unknown primary site'/exp OR 'cancer of unknown primary site' | 6,128 |

**Scopus**

| ( ( TITLE-ABS ( echocardiograp* OR holograph* OR "microwave tomograph*" OR "cell tracking*" OR neuroimaging* OR "brain cortical thickness" OR "brain mapping" OR neuroradiograph* OR "cerebral angiograph*" OR "cerebral ventriculograph*" OR echoencephalograph* OR myelograph* OR pneumoencophalograph* OR echoencephalograph* OR myelograph* OR photography OR photographies OR holography OR holographies OR photofluorograph* OR photogrammetr* OR "moire topograph*" OR "radiostereometric analys*" OR radiograph* OR "photon absorptiometry" OR x-ray* OR xray* OR "dxa scan*" OR "dexa scan*" OR angiograph* OR arthrograph* OR electrokymograph* OR fluoroscop* OR hysterosalpingograph* OR lymphograp* OR mammograph* OR microradiograph* OR neuroradiograph* OR pneumoradiograph* OR urograph* OR xeroradiograph* OR "radioisotope scan*" OR scintigraph* OR scintiphotograph* OR lymphoscintigraph* OR radioimmunodetection* OR "radiolabelled immunoscintigraph*" OR radioimmunoimaging* OR radioimmunoscintigraph* OR "radiolabeled immunoscintigraph*" OR "ventilation-perfusion scan*" OR "v-q scintigraph*" OR "vq lung scan*" OR "lung vq scan*" OR "near-infrared spectroscop*" OR "nir spectroscop*" OR "near-infrared spectrometr*" OR "nir spectrometr*" OR "subtraction technique*" OR thermograph* OR "temperature mapping*" OR echocardiograph* OR endosconograph* OR ultrasonograph* OR "whole body imaging*" OR ultrasound* OR "whole body scan*" OR "whole body screening*" ) OR AUTHKEY ( echocardiograp* OR holograph* OR "microwave tomograph*" OR "cell tracking*" OR neuroimaging* OR "brain cortical thickness" OR "brain mapping" OR neuroradiograph* OR "cerebral angiograph*" OR "cerebral ventriculograph*" OR echoencephalograph* OR myelograph* OR pneumoencophalograph* OR echoencephalograph* OR myelograph* OR photography OR photographies OR holography OR holographies OR photofluorograph* OR photogrammetr* OR "moire topograph*" OR "radiostereometric analys*" OR radiograph* OR "photon absorptiometry" OR x-ray* OR xray* OR "dxa scan*" OR "dexa scan*" OR angiograph* OR arthrograph* OR electrokymograph* OR fluoroscop* OR hysterosalpingograph* OR lymphograp* OR mammograph* OR microradiograph* OR neuroradiograph* OR pneumoradiograph* OR urograph* OR xeroradiograph* OR "radioisotope scan*" OR scintigraph* OR scintiphotograph* OR lymphoscintigraph* OR radioimmunodetection* OR "radiolabelled immunoscintigraph*" OR radioimmunoimaging* OR radioimmunoscintigraph* OR "radiolabeled immunoscintigraph*" OR "ventilation-perfusion scan*" OR "v-q scintigraph*" OR "vq lung scan*" OR "lung vq scan*" OR "near-infrared spectroscop*" OR "nir spectroscop*" OR "near-infrared spectrometr*" OR "nir spectrometr*" OR "subtraction technique*" OR thermograph* OR "temperature mapping*" OR echocardiograph* OR endosconograph* OR ultrasonograph* OR "whole body imaging*" OR ultrasound* OR "whole body scan*" OR "whole body screening*" ) ) OR ( TITLE-ABS ( "positron emission tomograph*" OR "petscan*" OR pet ) OR AUTHKEY ( "positron emission tomograph*" OR "petscan*" OR pet ) ) OR ( TITLE-ABS ( "magnetic resonance imag*" OR "nmr imag*" OR "mr tomograph*" OR mri OR mris OR fmri* OR "nmr tomograph*" OR zeugmatograph* OR "chemical shift imaging*" OR "magnetization transfer contrast imaging*" ) OR AUTHKEY ( "magnetic resonance imag*" OR "nmr imag*" OR "mr tomograph*" OR mri OR mris OR fmri* OR "nmr tomograph*" OR zeugmatograph* OR "chemical shift imaging*" OR "magnetization transfer contrast imaging*" ) ) OR ( TITLE-ABS ( "computed tomograph*" OR ct OR "computer assisted tomograph*" OR "computerized tomograph*" OR "CAT scan*" OR "electron beam tomograph*" OR "computerized axial tomograph*" OR spect* ) OR AUTHKEY ( "computed tomograph*" OR ct OR "computer assisted tomograph*" OR "computerized tomograph*" OR "CAT scan*" OR "electron beam tomograph*" OR "computerized axial tomograph*" OR spect* ) ) OR ( TITLE-ABS ( imaging* OR "computer-assisted image interpretation" OR "radiographic-assisted image interpretation" ) OR AUTHKEY ( imaging* OR "computer-assisted image interpretation" OR "radiographic-assisted image interpretation" ) ) ) AND ( TITLE-ABS ( primary W/3 ( occult OR unknown OR undefined OR hidden OR obscure OR discern ) W/6 ( cancer* OR neoplas* OR carcinoma* OR tumor* OR tumour* OR malignan* ) ) OR AUTHKEY ( primary W/3 ( occult OR unknown OR undefined OR hidden OR obscure OR discern ) W/6 ( cancer* OR neoplas* OR carcinoma* OR tumor* OR tumour* OR malignan* ) ) OR ( TITLE-ABS ( ( muo OR cup ) AND ( cancer* OR neoplas* OR carcinoma* OR tumor* OR tumour* OR malignan* ) ) OR AUTHKEY ( ( muo OR cup ) AND ( cancer* OR neoplas* OR carcinoma* OR tumor* OR tumour* OR malignan* ) ) ) OR ( TITLE-ABS ( "malignanc* of unknown origin" OR "cancer* of unknown origin" OR "neoplas* of unknown origin" OR "carcinoma* of unknown origin" OR "tumor* of unknown origin" OR "tumour* of unknown origin" ) OR AUTHKEY ( "malignanc* of unknown origin" OR "cancer* of unknown origin" OR "neoplas* of unknown origin" OR "carcinoma* of unknown origin" OR "tumor* of unknown origin" OR "tumour* of unknown origin" ) ) ) | 2436 |
| --- | --- |
