# Supplementary material for: Imaging of cancer of unknown primary: a systematic literature review of the past, present, and future
Source: Br J Radiol. 2025 Mar 21;98(1172):1209–26. doi: 10.1093/bjr/tqaf039 (PMC12341689; doi:10.1093/bjr/tqaf039)
Supplement: tqaf039_Supplementary_Data [file tqaf039_supplementary_data.zip › tqaf039_Supplementary_Data/CUP_Revision_Supplementary_TRACK_CHANGES.docx]

**Supplementary Material**

**Supplementary Table 1. Description of the search strategy**

**MEDLINE**

|  | **Search term** | **n_studies** |
| --- | --- | --- |
| **1** | **Neoplasms, Unknown Primary/** | **4064** |
| **2** | **((Occult or unknown or undefined or hidden or obscure or discern) adj3 primary adj6 (cancer* or neoplas* or carcinoma* or tumor* or tumor* or malignan*)).ti,ab,kf.** | **4346** |
| **3** | **((muo or cup) and (cancer* or neoplas* or carcinoma* or tumor* or tumour* or malignan*)).ti,ab,kf.** | **2141** |
| **4** | **("malignanc* of unknown origin" or "cancer* of unknown origin" or "neoplas* of unknown origin" or "carcinoma* of unknown origin" or "tumor* of unknown origin" or "tumor* of unknown origin").ti,ab,kf.** | **698** |
| **5** | **1 or 2 or 3 or 4** | **8186** |
| **6** | **exp diagnostic imaging/** | **2984617** |
| **7** | **(imaging* or "computer-assisted image interpretation" or "radiographic-assisted image interpretation").ti,ab,kf.** | **1139859** |
| **8** | **exp Tomography, X-Ray Computed/** | **507352** |
| **9** | **("computed tomograph*" or CT or "computer assisted tomograph*" or "computerized tomograph*" or "CAT scan*" or "electron beam tomograph*" or "computerized axial tomograph*" or spect*).ti,ab,kf.** | **2304757** |
| **10** | **exp Magnetic Resonance Imaging/** | **553817** |
| **11** | **("magnetic resonance imag*" or "nmr imag*" or "mr tomograph*" or mri or mris or fmri* or "nmr tomograph*" or zeugmatograph* or "chemical shift imaging*" or "magnetization transfer contrast imaging*").ti,ab,kf.** | **550555** |
| **12** | **exp Positron-Emission Tomography/** | **83762** |
| **13** | **("positron emission tomograph*" or "petscan*" or pet).ti,ab,kf.** | **162088** |
| **14** | **(echocardiograp* or holograph* or "microwave tomograph*" or "cell tracking*" or neuroimaging* or "brain cortical thickness" or "brain mapping" or neuroradiograph* or "cerebral angiograph*" or "cerebral ventriculograph*" or echoencephalograph* or myelograph* or pneumoencophalograph* or echoencephalograph* or myelograph* or photography or photographies or holography or holographies or photofluorograph* or photogrammetr* or "moire topograph*" or "radiostereometric analys*" or radiograph* or "photon absorptiometry" or x-ray* or xray* or "dxa scan*" or "dexa scan*" or angiograph* or arthrograph* or electrokymograph* or fluoroscop* or hysterosalpingograph* or lymphograp* or mammograph* or microradiograph* or neuroradiograph* or pneumoradiograph* or urograph* or xeroradiograph* or "radioisotope scan*" or scintigraph* or scintiphotograph* or lymphoscintigraph* or radioimmunodetection* or "radiolabelled immunoscintigraph*" or radioimmunoimaging* or radioimmunoscintigraph* or "radiolabeled immunoscintigraph*" or "ventilation-perfusion scan*" or "v-q scintigraph*" or "vq lung scan*" or "lung vq scan*" or "near-infrared spectroscop*" or "nir spectroscop*" or "near-infrared spectrometr*" or "nir spectrometr*" or "subtraction technique*" or thermograph* or "temperature mapping*" or echocardiograph* or endosconograph* or ultrasonograph* or "whole body imaging*" or ultrasound* or "whole body scan*" or "whole body screening*").ti,ab,kf.** | **1738560** |
| **15** | **6 or 7 or 8 or 9 or 10 or 11 or 12 or 13 or 14** | **5799783** |
| **16** | **5 and 15** | **3035** |

**Embase**

|  | **Query** | **Last results** |
| --- | --- | --- |
| **#17** | **#16 NOT ('conference abstract'/it OR 'conference paper'/it OR 'conference review'/it)** | **3,299** |
| **#16** | **#5 AND #15** | **4,912** |
| **#15** | **#6 OR #7 OR #8 OR #9 OR #10 OR #11 OR #12 OR #13 OR #14** | **6,291,458** |
| **#14** | **echocardiograp*:ti,ab,kw OR holograph*:ti,ab,kw OR 'microwave tomograph*':ti,ab,kw OR 'cell tracking*':ti,ab,kw OR neuroimaging*:ti,ab,kw OR 'brain cortical thickness':ti,ab,kw OR 'brain mapping':ti,ab,kw OR 'cerebral angiograph*':ti,ab,kw OR 'cerebral ventriculograph*':ti,ab,kw OR pneumoencophalograph*:ti,ab,kw OR echoencephalograph*:ti,ab,kw OR myelograph*:ti,ab,kw OR photography:ti,ab,kw OR photographies:ti,ab,kw OR holography:ti,ab,kw OR holographies:ti,ab,kw OR photofluorograph*:ti,ab,kw OR photogrammetr*:ti,ab,kw OR 'moire topograph*':ti,ab,kw OR 'radiostereometric analys*':ti,ab,kw OR radiograph*:ti,ab,kw OR 'photon absorptiometry':ti,ab,kw OR 'x ray*':ti,ab,kw OR xray*:ti,ab,kw OR 'dxa scan*':ti,ab,kw OR 'dexa scan*':ti,ab,kw OR angiograph*:ti,ab,kw OR arthrograph*:ti,ab,kw OR electrokymograph*:ti,ab,kw OR fluoroscop*:ti,ab,kw OR hysterosalpingograph*:ti,ab,kw OR lymphograp*:ti,ab,kw OR mammograph*:ti,ab,kw OR microradiograph*:ti,ab,kw OR neuroradiograph*:ti,ab,kw OR pneumoradiograph*:ti,ab,kw OR urograph*:ti,ab,kw OR xeroradiograph*:ti,ab,kw OR 'radioisotope scan*':ti,ab,kw OR scintigraph*:ti,ab,kw OR scintiphotograph*:ti,ab,kw OR lymphoscintigraph*:ti,ab,kw OR radioimmunodetection*:ti,ab,kw OR 'radiolabelled immunoscintigraph*':ti,ab,kw OR radioimmunoimaging*:ti,ab,kw OR radioimmunoscintigraph*:ti,ab,kw OR 'radiolabeled immunoscintigraph*':ti,ab,kw OR 'ventilation-perfusion scan*':ti,ab,kw OR 'v-q scintigraph*':ti,ab,kw OR 'vq lung scan*':ti,ab,kw OR 'lung vq scan*':ti,ab,kw OR 'near-infrared spectroscop*':ti,ab,kw OR 'nir spectroscop*':ti,ab,kw OR 'near-infrared spectrometr*':ti,ab,kw OR 'nir spectrometr*':ti,ab,kw OR 'subtraction technique*':ti,ab,kw OR thermograph*:ti,ab,kw OR 'temperature mapping*':ti,ab,kw OR echocardiograph*:ti,ab,kw OR endosconograph*:ti,ab,kw OR ultrasonograph*:ti,ab,kw OR 'whole body imaging*':ti,ab,kw OR ultrasound*:ti,ab,kw OR 'whole body scan*':ti,ab,kw OR 'whole body screening*':ti,ab,kw** | **2,353,848** |
| **#13** | **'positron emission tomograph*':ti,ab,kw OR 'petscan*':ti,ab,kw OR pet:ti,ab,kw** | **270,058** |
| **#12** | **'positron emission tomography'/exp** | **251,500** |
| **#11** | **'magnetic resonance imag*':ti,ab,kw OR 'nmr imag*':ti,ab,kw OR 'mr tomograph*':ti,ab,kw OR mri:ti,ab,kw OR mris:ti,ab,kw OR fmri*:ti,ab,kw OR 'nmr tomograph*':ti,ab,kw OR zeugmatograph*:ti,ab,kw OR 'chemical shift imaging*':ti,ab,kw OR 'magnetization transfer contrast imaging*':ti,ab,kw** | **855,107** |
| **#10** | **'nuclear magnetic resonance imaging'/exp** | **1,322,604** |
| **#9** | **'computed tomograph*':ti,ab,kw OR ct:ti,ab,kw OR 'computer assisted tomograph*':ti,ab,kw OR 'computerized tomograph*':ti,ab,kw OR 'cat scan*':ti,ab,kw OR 'electron beam tomograph*':ti,ab,kw OR 'computerized axial tomograph*':ti,ab,kw OR spect*:ti,ab,kw** | **2,910,614** |
| **#8** | **'x-ray computed tomography'/exp** | **114,012** |
| **#7** | **imaging*:ti,ab,kw OR 'computer-assisted image interpretation':ti,ab,kw OR 'radiographic-assisted image interpretation':ti,ab,kw** | **1,598,453** |
| **#6** | **'diagnostic imaging'/exp** | **275,836** |
| **#5** | **#1 OR #2 OR #3 OR #4** | **12,772** |
| **#4** | **malignanc* of unknown origin':ti,ab,kw OR 'cancer* of unknown origin':ti,ab,kw OR 'neoplas* of unknown origin':ti,ab,kw OR 'carcinoma* of unknown origin':ti,ab,kw OR 'tumor* of unknown origin':ti,ab,kw OR 'tumour* of unknown origin':ti,ab,kw** | **1,048** |
| **#3** | **(muo:ti,ab,kw OR cup:ti,ab,kw) AND (cancer*:ti,ab,kw OR neoplas*:ti,ab,kw OR carcinoma*:ti,ab,kw OR tumor*:ti,ab,kw OR tumour*:ti,ab,kw OR malignan*:ti,ab,kw)** | **3,819** |
| **#2** | **((occult OR unknown OR undefined OR hidden OR obscure OR discern) NEAR/3 primary NEAR/6 (cancer* OR neoplas* OR carcinoma* OR tumor* OR tumour* OR malignan*)):ti,ab,kw** | **6,992** |
| **#1** | **'cancer of unknown primary site'/exp OR 'cancer of unknown primary site'** | **6,128** |

**Scopus**

| **( ( TITLE-ABS ( echocardiograp* OR holograph* OR "microwave tomograph*" OR "cell tracking*" OR neuroimaging* OR "brain cortical thickness" OR "brain mapping" OR neuroradiograph* OR "cerebral angiograph*" OR "cerebral ventriculograph*" OR echoencephalograph* OR myelograph* OR pneumoencophalograph* OR echoencephalograph* OR myelograph* OR photography OR photographies OR holography OR holographies OR photofluorograph* OR photogrammetr* OR "moire topograph*" OR "radiostereometric analys*" OR radiograph* OR "photon absorptiometry" OR x-ray* OR xray* OR "dxa scan*" OR "dexa scan*" OR angiograph* OR arthrograph* OR electrokymograph* OR fluoroscop* OR hysterosalpingograph* OR lymphograp* OR mammograph* OR microradiograph* OR neuroradiograph* OR pneumoradiograph* OR urograph* OR xeroradiograph* OR "radioisotope scan*" OR scintigraph* OR scintiphotograph* OR lymphoscintigraph* OR radioimmunodetection* OR "radiolabelled immunoscintigraph*" OR radioimmunoimaging* OR radioimmunoscintigraph* OR "radiolabeled immunoscintigraph*" OR "ventilation-perfusion scan*" OR "v-q scintigraph*" OR "vq lung scan*" OR "lung vq scan*" OR "near-infrared spectroscop*" OR "nir spectroscop*" OR "near-infrared spectrometr*" OR "nir spectrometr*" OR "subtraction technique*" OR thermograph* OR "temperature mapping*" OR echocardiograph* OR endosconograph* OR ultrasonograph* OR "whole body imaging*" OR ultrasound* OR "whole body scan*" OR "whole body screening*" ) OR AUTHKEY ( echocardiograp* OR holograph* OR "microwave tomograph*" OR "cell tracking*" OR neuroimaging* OR "brain cortical thickness" OR "brain mapping" OR neuroradiograph* OR "cerebral angiograph*" OR "cerebral ventriculograph*" OR echoencephalograph* OR myelograph* OR pneumoencophalograph* OR echoencephalograph* OR myelograph* OR photography OR photographies OR holography OR holographies OR photofluorograph* OR photogrammetr* OR "moire topograph*" OR "radiostereometric analys*" OR radiograph* OR "photon absorptiometry" OR x-ray* OR xray* OR "dxa scan*" OR "dexa scan*" OR angiograph* OR arthrograph* OR electrokymograph* OR fluoroscop* OR hysterosalpingograph* OR lymphograp* OR mammograph* OR microradiograph* OR neuroradiograph* OR pneumoradiograph* OR urograph* OR xeroradiograph* OR "radioisotope scan*" OR scintigraph* OR scintiphotograph* OR lymphoscintigraph* OR radioimmunodetection* OR "radiolabelled immunoscintigraph*" OR radioimmunoimaging* OR radioimmunoscintigraph* OR "radiolabeled immunoscintigraph*" OR "ventilation-perfusion scan*" OR "v-q scintigraph*" OR "vq lung scan*" OR "lung vq scan*" OR "near-infrared spectroscop*" OR "nir spectroscop*" OR "near-infrared spectrometr*" OR "nir spectrometr*" OR "subtraction technique*" OR thermograph* OR "temperature mapping*" OR echocardiograph* OR endosconograph* OR ultrasonograph* OR "whole body imaging*" OR ultrasound* OR "whole body scan*" OR "whole body screening*" ) ) OR ( TITLE-ABS ( "positron emission tomograph*" OR "petscan*" OR pet ) OR AUTHKEY ( "positron emission tomograph*" OR "petscan*" OR pet ) ) OR ( TITLE-ABS ( "magnetic resonance imag*" OR "nmr imag*" OR "mr tomograph*" OR mri OR mris OR fmri* OR "nmr tomograph*" OR zeugmatograph* OR "chemical shift imaging*" OR "magnetization transfer contrast imaging*" ) OR AUTHKEY ( "magnetic resonance imag*" OR "nmr imag*" OR "mr tomograph*" OR mri OR mris OR fmri* OR "nmr tomograph*" OR zeugmatograph* OR "chemical shift imaging*" OR "magnetization transfer contrast imaging*" ) ) OR ( TITLE-ABS ( "computed tomograph*" OR ct OR "computer assisted tomograph*" OR "computerized tomograph*" OR "CAT scan*" OR "electron beam tomograph*" OR "computerized axial tomograph*" OR spect* ) OR AUTHKEY ( "computed tomograph*" OR ct OR "computer assisted tomograph*" OR "computerized tomograph*" OR "CAT scan*" OR "electron beam tomograph*" OR "computerized axial tomograph*" OR spect* ) ) OR ( TITLE-ABS ( imaging* OR "computer-assisted image interpretation" OR "radiographic-assisted image interpretation" ) OR AUTHKEY ( imaging* OR "computer-assisted image interpretation" OR "radiographic-assisted image interpretation" ) ) ) AND ( TITLE-ABS ( primary W/3 ( occult OR unknown OR undefined OR hidden OR obscure OR discern ) W/6 ( cancer* OR neoplas* OR carcinoma* OR tumor* OR tumour* OR malignan* ) ) OR AUTHKEY ( primary W/3 ( occult OR unknown OR undefined OR hidden OR obscure OR discern ) W/6 ( cancer* OR neoplas* OR carcinoma* OR tumor* OR tumour* OR malignan* ) ) OR ( TITLE-ABS ( ( muo OR cup ) AND ( cancer* OR neoplas* OR carcinoma* OR tumor* OR tumour* OR malignan* ) ) OR AUTHKEY ( ( muo OR cup ) AND ( cancer* OR neoplas* OR carcinoma* OR tumor* OR tumour* OR malignan* ) ) ) OR ( TITLE-ABS ( "malignanc* of unknown origin" OR "cancer* of unknown origin" OR "neoplas* of unknown origin" OR "carcinoma* of unknown origin" OR "tumor* of unknown origin" OR "tumour* of unknown origin" ) OR AUTHKEY ( "malignanc* of unknown origin" OR "cancer* of unknown origin" OR "neoplas* of unknown origin" OR "carcinoma* of unknown origin" OR "tumor* of unknown origin" OR "tumour* of unknown origin" ) ) )** | **2436** |
| --- | --- |
